# Reporting of context and implementation in studies of global health interventions: a pilot study

**DOI:** 10.1186/1748-5908-9-57

**Published:** 2014-05-12

**Authors:** Jill Luoto, Paul G Shekelle, Margaret A Maglione, Breanne Johnsen, Tanja Perry

**Affiliations:** 1 RAND Corporation, 1776 Main Street, Santa Monica CA, USA; 2 West Los Angeles Veterans Affairs Medical Center, Los Angeles CA, USA

## Abstract

**Background:**

There is an increasing push for ‘evidence-based’ decision making in global health policy circles. However, at present there are no agreed upon standards or guidelines for how to evaluate evidence in global health. Recent evaluations of existing evidence frameworks that could serve such a purpose have identified details of program context and project implementation as missing components needed to inform policy. We performed a pilot study to assess the current state of reporting of context and implementation in studies of global health interventions.

**Methods:**

We identified three existing criteria sets for implementation reporting and selected from them 10 criteria potentially relevant to the needs of policy makers in global health contexts. We applied these 10 criteria to 15 articles included in the evidence base for three global health interventions chosen to represent a diverse set of advocated global health programs or interventions: household water chlorination, prevention of mother-to-child transmission of HIV, and lay community health workers to reduce child mortality. We used a good-fair-poor/none scale for the ratings.

**Results:**

The proportion of criteria for which reporting was poor/none ranged from 11% to 54% with an average of 30%. Eight articles had ‘good’ or ‘fair’ documentation for greater than 75% of criteria, while five articles had ‘poor or none’ documentation for 50% of criteria or more. Examples of good reporting were identified.

**Conclusions:**

Reporting of context and implementation information in studies of global health interventions is mostly fair or poor, and highly variable. The idiosyncratic variability in reporting indicates that global health investigators need more guidance about what aspects of context and implementation to measure and how to report them. This lack of context and implementation information is a major gap in the evidence needed by global health policy makers to reach decisions.

## Background

Policy makers in global health are increasingly adopting ‘evidence-based’ decision making practices [1,2]. By using the available evidence to inform their decision making, it is believed that resultant policy choices can improve in terms of their appropriateness in being applied to a particular context, or likelihood of achieving their envisioned aims. However, at present there are no commonly accepted guidelines within global public health for how to evaluate evidence. Evaluations of existing evidence frameworks have identified details of program context and project implementation as needed, yet missing, components [3,4]. In this pilot study, we evaluated how context and implementation were reported in recent studies of global health interventions in order to identify areas reported sufficiently well, and areas where action is needed to improve the design, conduct, and reporting of global health interventions to better enable decision-makers to make sound, evidence-based decisions.

## Methods

In this study, we identified candidate criteria for reporting context and implementation, selected a representative sample of published global health intervention studies, and then applied the candidate criteria to the published studies. To assist us in the first two tasks, we assembled an international and multidisciplinary Technical Expert Panel (TEP) consisting of intervention developers, evaluators, practitioners, sponsors and policy makers regarding global health (see acknowledgements for list of technical experts, totaling 17 in number). Experts were identified based on publication records covering global health interventions or program evaluation, as well as suggestions from policymaking organizations and sponsors. This was the second phase of a study assessing the utility of existing global health evidence frameworks, the first phase of which has already been published. That study found that existing evidence frameworks vary in their criteria and judgments, and do not sufficiently take into account all of the many needs of decision-makers in global health contexts [3].

### Identifying candidate criteria

As part of the discussion of the utility of existing global health evidence frameworks, the TEP identified information about context and implementation as gaps in current evidence frameworks. More specifically, they identified the need for more information about who delivered the intervention, what the intervention consisted of, how it was implemented, what it cost, and contextual details about the population receiving the intervention. We used this input, plus our own knowledge of implementation science, to guide our selection of candidate criteria for reporting of context and implementation. We chose to use existing criteria, selected according to their applicability to the needs identified by the TEP. We used three existing sets of implementation criteria (IC) identified by experts in implementation science: the Consolidated Framework for Implementation Research (CFIR [5]); the proposed criteria for reporting the development and evaluation of complex interventions in healthcare (CReDECI [6]); and those criteria required by the editors of the journal Implementation Science, themselves based on the WIDER criteria [7]. From these IC sets, we identified specific criteria based on the input described above. We tried to include at least one criterion from each of the five domains in the Consolidated Framework for Implementation Research: intervention characteristics, outer setting, inner setting, characteristics of the individuals involved, and the process of implementation. We ended up selecting 10 implementation criteria as a parsimonious set that were potentially relevant to report for implementation of global health interventions, and therefore worth testing in a pilot study. One criterion had eight components that were each rated separately.

### Identifying a representative sample of published global health intervention studies

We identified a diverse set of global health interventions as potential candidates with which to apply these existing implementation criteria by considering the major causes of morbidity and mortality in developing countries or the major diseases of focus among international global health financing bodies. This set of global health interventions was chosen both to apply the selected implementation criteria as well as existing evidence frameworks as part of the first phase of this study (described elsewhere) [3].

We developed a draft set of key dimensions for classifying global health interventions in order to map out these potential exemplar interventions to select a diverse set along these dimensions (*e.g*., population affected, geographic location, whether the intervention addresses a communicable or non-communicable disease, whether the target is a one-time behavior change or recurring event, etc.). TEP members provided input on the dimensions and on their preferred exemplars. From this exercise, we selected three interventions: household water chlorination, prevention of mother-to-child transmission of HIV (PMTCT), and lay or community health workers to reduce childhood morbidity and mortality. Details of this selection process are presented in more detail elsewhere [8].

For each of the three chosen representative global health interventions, we located published systematic reviews of their effectiveness by conducting a Medline search and selected one review for use in this pilot study. The reviews chosen for each intervention were the most recent ones that we judged best assessed the representative interventions. For each of these reviews, we retrieved the original research studies included in the review and used these original studies as sources of evidence when applying the pilot implementation criteria.

### Applying the criteria

For each of the 10 implementation criteria and each of the original research articles used in the systematic reviews on each of the representative global health interventions, one reviewer recorded the exact text that was judged related to the criterion, and assigned an initial score of ‘good,’ ‘fair,’ ‘poor/none’ following a rating scheme used by many other quality and reporting checklists. Then, at a group meeting, each criterion for each article was reviewed in detail, and a group decision was reached regarding the final rating, based on the degree to which we judged the text met the needs of stakeholders regarding that aspect of implementation, as determined by the input received from our technical expert panel.

## Results and discussion

### Results

Table 1 lists the 10 implementation criteria adapted for this project from the 3 IC sets. The provided text examples accompanying these criteria are ones we identified from the global health research articles. The rationale or clarifying statements are taken directly from the original source, with adaptation to the global health context.

**Table 1:** Global framework – implementation criteria for pilot test

**Criterion**	**Source**	**Rationale or clarifying statement**	**Example**
**Criterion #1** - Intervention characteristics: Intervention/Program source	CFIR, Damschroder, [5]	Is the intervention/program externally or internally developed? An intervention/program may be internally developed as a good idea, a solution to a problem, or other grass roots effort, or may be developed by an external entity (such as a foundation or a NGO). Interventions or programs that arise internally from the populations who will be impacted are sometimes more sustainable than externally developed programs dependent on external funding. The perceived legitimacy of the source may also influence implementation.	‘The ViSION project involved a partnership among Save the Children/US ([SC] Hanoi and Westport, Conn., USA), the USAID-funded LINKAGE Project (Washington, D.C.), Emory University Rollins School of Public Health (Atlanta, Ga., USA), and the Research and Training Center for Community Development (RTCCD, Hanoi). The SC Viet Nam field office developed the program model and implemented it through government partners. (Indicates this is externally funded).’
**Criterion #2** - Intervention characteristics: A description of why the intervention was hypothesized to have an impact on the outcome, according to theory.	CReDECI, Mohler also mentioned in Michie, [6,7]	The theoretical basis of the intervention should be clearly stated. This includes the theory on which the intervention is founded as well as, if available, empirical evidence from studies in different settings or countries.	‘Previously, we showed no effect of direct education by health workers on infant care practices and care-seeking behavior after delivery. In view of the Bolivian model, we thought that a participatory approach might have more effect on perinatal care practices and might increase consultation for difficulties in pregnancy and the newborn period. Two key elements distinguished our approach from conventional health education. First, women’s groups looked at demand-side and supply-side issues. Second, the approach emphasized participatory learning rather than instruction.’
**Criterion #3** - Intervention characteristics: Rationale for the aim/essential functions of the intervention/program’s components, including the evidence whether the components are appropriate for achieving this goal.	CReDECI, Mohler, also mentioned in Michie, [6,7]	This differs from the need to articulate the theory behind the intervention in that the theory posits the general principles (such as Rogers Diffusion of Innovation) while this item is about specific components of the intervention and the effects of the component on specific targets.	‘Our preliminary qualitative field work showed that individual behaviours were influenced by collective behaviours and social norms, and sustained by a complex, multilevel network of relationships within the community. We therefore developed a multilevel strategy targeting: community stakeholders, newborn stake holders, and households with immediate support groups. At each level, the target group consisted of individuals who were identified to have key roles as influencers, decision-makers, supporters, and practitioners of newborn care and normative behaviour within the community. The support of community stake holders such as village heads, community leaders, respected members, priests, and teachers was crucial in building trust with the community and ensuring acceptance of the program. The newborn stakeholder target group included traditional newborn-care providers and birth attendants, unqualified medical practitioners, and, to a lesser extent, health system workers, some of whom had strategic access to the newborn and mother during post-partum confinement, were perceived by the community as domain experts, and played an active part in sustaining targeted practices. Health system workers such as auxiliary nurse midwives were engaged only at the community level as part of newborn stakeholder group meetings in order to keep contamination of the intervention into control clusters to a minimum. The household target group included the pregnant woman or mother, who was the primary care provider, but usually not empowered to make decisions; the mother-in-law, who was usually the key decision maker on newborn-care practices; other female members who played supportive roles; and male members, including the father-in-law and husband, who controlled access to the household, made financial and logistical arrangements, and influenced care-seeking decisions. The family’s immediate support group included neighbors and relatives who influenced family behaviors and helped with deliveries.’
**Criterion #4** - Intervention Characteristics: Detailed description of the intervention/program	WIDER as described in Michie, [7]	None beyond those stated in each criterion.	None.
**The detailed description should include:**			
a. Characteristics of those delivering the intervention/program (such as a nurse or lay health worker)			
b. Characteristics of the recipients			
c. The setting			
d. The mode of delivery (such as face-to-face)			
e. The intensity of the intervention/program (such as the contact time with participants)			
f. The duration (such as the number of sessions and their spacing interval over a given period)			
g. Adherence or fidelity to delivery protocols			
h. A detailed description of the intervention/program content provided to each study group			
**Criterion #5** - Intervention Characteristics: Costs of the intervention and costs associated with implementing the intervention	CFIR, Damschroder, CReDECI, Mohler, [5,7]	The cost of the intervention and implementation can influence the adoption and sustainability; interventions may be more difficult to sustain if they were supported as part of a research study.	No good reporting examples were identified.The closest examples of good cost reporting mentioned cost without stating whether it was program or research.
**Criterion #6** - Outer Setting: External policies and incentives	CFIR, Damschroder, [5]	How does the health service, intervention, or program relate to country and global health goals? Is the program part of a larger strategy? If so how is it strategically aligned? A country's health policies may influence the implementation of a particular intervention or program.	We found we could not operationalize this criterion, and hence, identified no examples of good reporting
**Criterion #7** - Population needs	CFIR, Damschroder, [5]	The extent to which population needs, as well as barriers and facilitators to meet those needs, are accurately known and prioritized. This could include population-based data on causes of morbidity and mortality, political or cultural barriers or facilitators, and/or more locally focused data about local needs, barriers or facilitators.	‘In these communities, infant mortality is high, and 40% of all deaths among children less than five years of age are due to diarrhea. In prior studies, households in these communities that added dilute bleach to their highly contaminated drinking water and stored it in vessels that prevented recontamination had markedly less contaminated water than households with standard water handling practices.’
**Criterion #8** - Process of implementation: Description of facilitators or barriers which have influenced the intervention or program’s implementation (see #10) revealed by a process assessment.	CReDECI, Mohler, also mentioned in Michie, [6,7]	In contrast to the criterion #7 above which assesses barriers and facilitators as inputs to developing the intervention strategy, this criterion assesses the actual barriers and facilitators identified during and after the implementation.	‘The reasons cited for non-compliance (multiple responses allowed) included: nobody was available to accompany the child (and the mother) to the health facility (24.7%); the child was given a traditional treatment instead (191%); bad weather or general strikes (17.9%); the family disliked hospital treatment (12.3%); symptoms resolved on their own (7.4%); unwillingness of the family or the TBA to refer the baby for other reasons (6.2%); and other issues (12.3%), such as illness of the mother; the child was too young to be taken for outside care; and lack of transport.
			Substantial increases in referral compliance for newborn illness were likely related to (a) education of families on danger signs by the CHWs; (b) active surveillance for illness by the CHWs during routine postnatal home-visits; (c) facilitated referral by the CHWs, including counselling, use of referral slips along with improved linkages between community and hospital; (d) incentives for labour/birth notification; (e) enhanced capacity at the referral –care center to manage sick newborns; and (f) availability of subsidized treatment. Sustained community-level education enhanced the empowerment of families towards decision-making for self-referral.’
**Criterion #9** - Description of materials: Description of all materials or tools used for the implementation	CReDECI, Mohler, [6]	This refers to printed materials, videos, pictures, syllabi, etc. used for training or implementation	The research study references a five volume field training manual.
**Criterion #10** - Process of Implementation: Description of an assessment of the implementation process	CReDECI, Mohler [6]	Process assessment is a prerequisite for determining the success of the intervention's implementation and should be an integral part of an assessment of the intervention’s effect.	‘To gain insight into the dissemination and the delivery of the intervention and to draw conclusions about potential barriers and facilitators to implementing the intervention in other settings, data on the implementation process were collected alongside the randomized-controlled trial. Therefore, we assessed the quality of delivery of the interventional components (observed by members of the research team not involved in the delivery of the intervention) and the adherence to study protocol (number and type of deviations from the protocol, using a pilot-tested standardized form). We also analyzed barriers and facilitators for the delivery of intervention’s components (focus group interviews with intervention participants).’

For the pilot testing of these implementation criteria, we applied the 10 implementation criteria in Table 1 to the 15 original research articles that form the evidence base for our three global health interventions. For the exemplar intervention of household water chlorination, we used the three original household chlorination research studies that were included in the Clasen *et al*. [9] meta-analysis for the outcome of rate-ratios for all-age diarrhea (see Analysis 1.1 stratified by intervention type). Furthermore, we include the published journal version for one of these studies instead of the original dissertation, both due to practical convenience and because this is likely to be what would be available to a policymaker undertaking a similar exercise (we substitute the published version of Sobsey *et al*. [10] for the original dissertation credit of Handzel [11]). For the representative intervention of PMTCT, we utilized five original research studies included in a 2012 systematic review on community strategies to improve PMTCT programs in the developing world [12]. The primary outcomes were prevention of vertical transmission of HIV as well as program retention. For the intervention of community or lay health workers to reduce child mortality, we utilized all seven trials contributing to the meta-analysis in the sole 2010 Cochrane review on this subject [13]. Thus, we applied 10 criteria to each of 15 articles.

Tables 2 and 3 present summary findings for household water chlorination, prevention of mother-to-child-transmission of HIV, and lay health workers by criterion and by article, respectively. Additional file 1 summarizes findings by article and by criterion together. More detailed tables assessing what text was found and how we judged it meeting the criteria can be found in Additional file 2.

**Table 2:** Ratings of implementation criteria in published studies of three representative global health interventions

**Criteria**	**3 Studies of household water chlorination**			**5 Studies of preventing mother-to-child transmission**			**7 Studies of lay/community health workers**			**Overall**		
	**(Number of articles reporting criterion)**			**(Number of articles reporting criterion)**			**(Number of articles reporting criterion)**			**(Number of articles reporting criterion)**		
	**Good**	**Fair**	**Poor/none**	**Good**	**Fair**	**Poor/none**	**Good**	**Fair**	**Poor/none**	**Good**	**Fair**	**Poor/none**
1.	3	0	0	4	1	0	6	1	0	13	2	0
2.	0	0	3	1	4	0	4	0	3	5	4	6
3.	0	0	3	0	4	1	2	2	3	2	6	7
4a.	1	0	2	3	2	0	3	3	1	7	5	3
4b.	1	1	1	4	1	0	4	2	1	9	4	2
4c.	2	1	0	2	3	0	6	1	0	10	5	0
4d.	3	0	0	5	0	0	4	3	0	12	3	0
4e.	0	2	1	1	4	0	4	1	2	5	7	3
4f.	1	2	0	4	0	1	5	0	2	10	2	3
4g.	0	0	3	4	0	1	3	3	1	7	3	5
4h.	0	0	3	4	1	0	2	1	4	6	2	7
5.	0	1	2	0	0	5	0	2	5	0	3	12
7.	1	2	0	3	2	0	3	2	2	7	6	2
8.	0	1	2	4	1	0	2	1	4	6	3	6
9.	0	0	3	3	2	0	2	1	4	5	3	7
10.	0	0	3	1	3	1	3	0	4	4	3	8
**Totals**	**12**	**10**	**26**	**43**	**28**	**9**	**53**	**23**	**36**	**108**	**61**	**71**
**Proportion of articles reporting (%)**	**25%**	**21%**	**54%**	**54%**	**35%**	**11%**	**47%**	**21%**	**32%**	**45%**	**25%**	**30%**

**Table 3:** Ratings of implementation criteria by article

	**Good**	**Fair**	**Poor/none**
**Household water chlorination**			
Lule *et al*.; [14]	2	5	9
Luby *et al*.; [15]	6	3	7
Sobsey *et al*.; [10]	4	2	10
**Preventing mother-to-child transmission**			
Futterman *et al*.; [16]	8	7	1
Torpey *et al*.; [17]	8	7	1
Farquhar *et al*.; [18]	8	7	1
Chandisarewa *et al*.; [19]	12	2	2
Bekker *et al*.; [20]	7	5	4
**Lay health workers**			
Sloan *et al*.; [21]	6	2	8
Kouyate *et al*.; [22]	2	6	8
Kumar *et al*.; [23]	12	1	3
Bari *et al*.; [24]	6	6	4
Chongsuvivatwong *et al*.; [25]	4	4	8
Manandhar *et al*.; [26]	9	3	4
Marsh *et al*.; [27,28]	14	1	1

We found we could not operationalize criterion 6, about the outer setting, and therefore dropped it. The remaining 9 criteria generated 16 ratings, since criterion 4 includes 8 subcriteria. The proportion of criteria for which reporting was poor or none ranged from 11% to 54% with an average of 30%. The two most common criteria for which reporting was rated poor or none were criteria 5 and 10, which dealt with, respectively, cost (either intervention or implementation) and describing or assessing the implementation process itself, in terms of the function or aims of each of the program components. The three most common criteria for which reporting was rated good were criteria 1, 4c, and 4d, which described the source of the intervention (in almost all cases, these were investigator initiated research projects), the setting, and the mode of delivery (such as face-to-face), respectively.

## Discussion

The three most important findings of this pilot study are: overall reporting is poor or none for about one third of a sample of criteria; the quality of reporting varies across articles and interventions; and good reporting is possible, with examples of good reporting for each criterion (except costs).

The reporting of implementation information is highly variable both within and across articles, with some articles reporting a great deal of information about some criteria and almost nothing about others, and likewise some articles report almost nothing about most criteria while others report a great deal about most criteria. For example, the articles by Chandisarewa [19], Kumar [23], and Marsh [28] are examples of good reporting on most criteria. In total, eight articles had ‘good’ or ‘fair’ documentation for greater than 75% of criteria, while five articles had ‘poor or none’ documentation for 50% or more criteria. This degree of variability within and across studies suggests that the decisions by each global health study team about which aspects of context and implementation to measure and report are idiosyncratic and not guided by any commonly accepted norms. This contrasts with the more standardized reporting of other aspects of study design and execution, such as whether or not a study uses random assignment to intervention groups and what the attrition rate may be for study participants. Reporting tools such as the CONSORT statement have helped improve the reporting of features such as these [29,30], and perhaps reporting of global health interventions could be similarly improved by the development and widespread adoption of reporting criteria.

In this pilot study, we made some observations that may prove useful to future assessments of context and implementation in global health interventions. First, as already noted, we found we could not satisfactorily operationalize the CFIR criterion about the outer setting, since we judged that in most or all of the settings the national and local health authorities would judge the aims of the interventions (*e.g*., reduce water-borne diarrheal illness, reduce mother-to-child transmission of HIV, reduce infant mortality) to be compatible with their health policies. Scoring this criterion as ‘poor or none’ if there was not an explicit statement in the article about this alignment of the intervention with health goals seemed too harsh, and hence we dropped this criterion. Secondly, because we used criteria from different sources, there were some that overlapped and could be consolidated; for example, criterion 4h, ‘A detailed description of the intervention/program content provided to each study group,’ and criterion 9, ‘Description of all materials or tools used for the implementation.’ Thirdly, we followed the common practice of giving the ‘benefit of the doubt’ when making judgments between categories, meaning our ratings are probably a ‘best case scenario’ for current reporting. More work in making sharper the operational definitions between different categories of reporting will be needed to avoid this upward bias. Lastly, we found some criteria more difficult to rate than others; specifically, criteria 2, 3, 7 and 8 were particularly difficult to judge, and we believe further work is needed to assess and improve inter-rater reliability. Our judgment is that the items 4a to 4h (from the WIDER criteria) are potentially the most immediately useful and applicable, with the addition of criterion 5 about costs, as we found these easiest to rate.

In summary, this pilot study found that reporting of context and implementation information in studies of global health interventions is at best mostly fair or poor, and highly variable. The lack of context and implementation information is a major gap in the evidence needed by global health policy makers to reach decisions. The idiosyncratic variability in reporting indicates global health investigators need more guidance about what aspects of context and implementation to measure and how to report them. This pilot study could be useful to an effort to develop that guidance. Without better reporting, policy makers will be left in the dark about context and implementation details that are key to designing and introducing an effective and sustainable intervention.

## Abbreviations

CFIR: Consolidated Framework for Implementation Research; CONSORT: Consolidated Standards of Reporting Trials; CReDECI: Criteria for reporting the development and evaluation of complex interventions in healthcare; HIV: Human immunodeficiency virus; IC: Implementation criteria; NGO: Non-governmental organization; PMTCT: Prevention of mother-to-child transmission of HIV; TEP: Technical expert panel; WIDER: Workgroup for Intervention Development and Evaluation Research.

## Competing interests

The authors declare no competing interests. This article is based on research conducted by the Southern California Evidence-Based Practice Center under contract with the Agency for Healthcare Research and Quality http://www.ahrq.gov (AHRQ; contract No. HHSA290200710062I). The authors of this article are responsible for its contents. No statement in this article should be construed as an official position of AHRQ or the US Department of Health and Human Services. The funder had no role in the study design, data collection and analysis, or decision to publish.

## Authors’ contributions

Analyzed the data: JL, MAM, PGS. Wrote the first draft of the manuscript: JL. Contributed to the writing of the manuscript: JL, MAM, PGS. Agree with manuscript results and conclusions: JL, MAM, BJ, TP, PGS. Obtained funding: PGS. Administrative, technical or material support: BJ, TP. Critical revision of the manuscript for important intellectual content: JL, MAM, PGS. Study supervision: PGS. All authors read and approved the final manuscript.

## Authors’ information

JL (PhD) is an Economist at the RAND Corporation. PS (MD, PhD) is an Internist who serves as the Chief of General Internal Medicine at the West Los Angeles Veterans Affairs Medical Center in Los Angeles. He is also the Director of the Southern California Evidence-based Practice Center based at the RAND Corporation in Santa Monica. MM (MPP) is the Associate Director of the Southern California Evidence-based Practice Center based at the RAND Corporation in Santa Monica. BJ (BS) was a Research Assistant and TP (BHM) was a Project Assistant. Both worked for the Southern California Evidence-Based Practice Center based at the RAND Corporation in Santa Monica.

## Supplementary Material

Additional file 1 **Ratings of implementation criteria in published studies of three representative Global Health Interventions, by criterion and by article.** The table presents the criteria ratings across all studies presented for Household Water Chlorination, Preventing Mother-to-Child Transmission of HIV, and Lay or Community Health Workers to reduce child mortality. Column 1 indicates the articles, and columns 2–11 indicate the criterion by which the article was assessed with a rating of good, fair, or poor/none. Click here for file

Additional file 2 **Detailed Criteria on Published Studies for each of 3 Exemplar Global Health Interventions.** Additional file 2 contains 15 tables presenting the results of each study across each criterion. Click here for file
