# Supplementary material for: Reporting of context and implementation in studies of global health interventions: a pilot study
Source: Implement Sci. 2014 May 12;9:57. doi: 10.1186/1748-5908-9-57 (PMC4043974; doi:10.1186/1748-5908-9-57)
Supplement: Additional file 1 — Ratings of implementation criteria in published studies of three representative Global Health Interventions, by criterion and by article. The table presents the criteria ratings across all studies presented for Household Water Chlorination, Preventing Mother-to-Child Transmission of HIV, and Lay or Community Health Workers to reduce child mortality. Column 1 indicates the articles, and columns 2–11 indicate the criterion by which the article was assessed with a rating of good, fair, or poor/none. [file 1748-5908-9-57-S1.doc]

**Additional File 1**. Ratings of implementation criteria in published studies of three representative Global Health Interventions, by criterion and by article

| Article | 1 | 2 | 3 | 4a | 4b | 4c | 4d | 4e | 4f | 4g | 4h | 5 | 6 | 7 | 8 | 9 | 10 |
| --- | --- | --- | --- | --- | --- | --- | --- | --- | --- | --- | --- | --- | --- | --- | --- | --- | --- |
| **Household Water Chlorination** | | | | | | | | | | | | | | | | | |
| Lule et al.; 2005 | Good | Poor | Poor | Poor | Fair | Fair | Good | Fair | Fair | Poor | Poor | Poor | * | Fair | Poor | Poor | Poor |
| Luby et al.; 2004 | Good | Poor | Poor | Good | Good | Good | Good | Fair | Fair | Poor | Poor | Fair | * | Good | Poor | Poor | Poor |
| Sobsey et al.; 2003 | Good | Poor | Poor | Poor | Poor | Good | Good | Poor | Good | Poor | Poor | Poor | * | Fair | Fair | Poor | Poor |
| **Preventing Mother-to-Child Transmission** | | | | | | | | | | | | | | | | | |
| Futterman; 2010 | Fair | Good | Fair | Good | Good | Fair | Good | Fair | Good | Good | Good | Poor | * | Good | Fair | Fair | Fair |
| Torpey; 2010 | Good | Fair | Fair | Fair | Good | Fair | Good | Fair | Good | Good | Good | Poor | * | Fair | Good | Good | Fair |
| Farquhar; 2004 | Good | Fair | Fair | Fair | Good | Fair | Good | Fair | Good | Good | Good | Poor | * | Fair | Good | Good | Fair |
| Chandisarewa; 2007 | Good | Fair | Fair | Good | Good | Good | Good | Good | Poor | Good | Good | Poor | * | Good | Good | Good | Good |
| Bekker; 2006 | Good | Fair | Poor | Good | Fair | Good | Good | Fair | Good | Poor | Fair | Poor | * | Good | Good | Fair | Poor |
| **Lay Health Workers** | | | | | | | | | | | | | | | | | |
| Sloan; 2008 | Good | Poor | Poor | Fair | Good | Good | Fair | Poor | Poor | Good | Poor | Poor | * | Poor | Good | Poor | Good |
| Kouyate; 2008 | Good | Poor | Poor | Fair | Fair | Good | Fair | Poor | Poor | Fair | Fair | Poor | * | Poor | Poor | Fair | Poor |
| Kumar; 2008 | Fair | Good | Good | Good | Good | Good | Good | Good | Good | Good | Good | Poor | * | Good | Poor | Good | Poor |
| Bari et al.; 2006 | Good | Good | Fair | Good | Poor | Fair | Fair | Good | Good | Fair | Poor | Poor | * | Fair | Fair | Poor | Good |
| Chongsuvivatwong et al.; 1996 | Good | Poor | Poor | Fair | Fair | Good | Good | Fair | Good | Poor | Poor | Poor | * | Fair | Poor | Poor | Poor |
| Manandhar et al.; 2004 | Good | Good | Fair | Good | Good | Good | Good | Good | Good | Fair | Poor | Fair | * | Good | Poor | Poor | Poor |
| Marsh et al.; 2002[28] | Good | Good | Good | Poor | Good | Good | Good | Good | Good | Good | Good | Fair | * | Good | Good | Good | Good |

* This domain was dropped after determining that scoring was too imprecise
