# Supplementary material for: Reporting of context and implementation in studies of global health interventions: a pilot study
Source: Implement Sci. 2014 May 12;9:57. doi: 10.1186/1748-5908-9-57 (PMC4043974; doi:10.1186/1748-5908-9-57)
Supplement: Additional file 2 — Detailed Criteria on Published Studies for each of 3 Exemplar Global Health Interventions. Additional file 2 contains 15 tables presenting the results of each study across each criterion. [file 1748-5908-9-57-S2.doc]

**Additional File 2. Detailed Criteria Ratings on Published Studies for each of 3 Global Health Exemplar Interventions.**

**Household Water Chlorination**

**Additional File 2-A. - Criterion Table for Luby et al.; 2004**

| **Criterion** | **Example of text related to this criterion** | **Rating** |
| --- | --- | --- |
| **Criterion #1**  **Intervention Characteristics:** Intervention/Program source  (From CFIR, Damschroder, 2009)  **Explanation/Example:**  Is the intervention/program externally or internally developed? An intervention/program may be internally developed as a good idea, a solution to a problem, or other grass roots effort, or may be developed by an external entity (such as a foundation or a NGO). Interventions or programs that arise internally from the populations who will be impacted are sometimes more sustainable than externally developed programs dependent on external funding. The perceived legitimacy of the source may also influence implementation. | Two home-based interventions, disinfecting water with bleach and washing hands with soap, have been proven in multiple small-scale evaluations to decrease the incidence of diarrhea. *(Indicates the intervention was developed externally)* | Good |
| **Criterion #2**  **Intervention Characteristics:** A description of why the intervention was hypothesized to have an impact on the outcome, according to theory. (From CReDECI, Mohler 2012; also mentioned in Michie, 2009)  **Explanation/Example:**  The theoretical basis of the intervention should be clearly stated. This includes the theory on which the intervention is founded as well as, if available, empirical evidence from studies in different settings or countries. For example, "The implementation was based on Rogers’ Diffusion of Innovation theory, which posits 5 factors of innovation that influence a decision to adopt or reject an innovation: relative advantage, compatibility, complexity or simplicity, trialability, observability. A similar intervention, also based on Rogers’ Diffusion of Innovation theory, was successfully implemented in other countries." | Field workers arranged neighborhood meetings and used slide shows, videotapes, and pamphlets to illustrate health problems resulting from hand and water contamination and to provide specific instructions on how to use the study intervention. Field workers visited each participating house at least weekly. In visits to intervention households, they promoted discussion and answered questions about the intervention, re-supplied families with bleach or soap, and encouraged regular use of the interventions. | Poor |
| **Criterion #3**  **Intervention Characteristics:**  Rationale for the aim/essential functions of the intervention/program’s components, including the evidence whether the components are appropriate for achieving this goal.  This differs from the need to articulate the theory behind the intervention in that the theory posits the general principles (such as Rogers Diffusion of Innovation) while this item is about specific components of the intervention and the effects of the component on specific targets.  (From CReDECI, Mohler, 2012; also mentioned in Michie, 2009) | “Two home-based interventions, disinfecting water with bleach and washing hands with soap, have been proven in multiple small-scale evaluations to decrease the incidence of diarrhea.” | Poor/none |
| **Criterion #4**  **Intervention Characteristics:**  Detailed description of the intervention/program (From WIDER as described in Michie, 2009)  **The detailed description should include:**  a. Characteristics of those delivering the intervention/program (such as a nurse or lay health worker)  b. Characteristics of the recipients  c. The setting  d. The mode of delivery (such as face-to-face)  e. The intensity of the intervention/program (such as the contact time with participants)  f. The duration (such as the number of sessions and their spacing interval over a given period)  g. Adherence or fidelity to delivery protocols  h. A detailed description of the intervention/program content provided to each study group | a. HOPE field workers who have completed at least 8 years of education and represented a variety of ethnic and linguistic groups and lived either in the study communities or nearby communities.  b. The population is described in detail in the introduction and in Table 1.  c. The setting and the characteristics are very similar if not identical in this article. The setting is three squatter settlements in central Karachi, Pakistan where only some households have access to municipal water supply system.  d. The intervention is delivered face-to-face. “Field workers arranged neighborhood meetings and used slide shows, videotapes, and pamphlets to illustrate helath problems resulting from hand and water contamination and to provide specific instructions on how to use the study intervention. Field workers visited each participating house at least weekly. In visits to intervention households, they promoted discussion and answered questions about the intervention, re-supplied families with bleach or soap, and encouraged regular use of the interventions”  e. The actual length of visits is never mentioned, but the number of visits is described in quote above. Plus the measurements section “Trained field workers conducted a pre-intervention baseline survey …..”  f. This might be answered by e. above.  g. No mention of this by HOPE workers tasked with implementing the intervention.  h. The quote above about videos and encouragement are quite generic. | Good  Good  Good  Good  Fair  Fair  Poor  Poor |
| **Criterion #5**  **Intervention Characteristics:**  Costs of the intervention and costs associated with implementing the intervention (From CFIR, Damschroder, 2009; CReDECI, Mohler, 2012)  **Explanation/Example:**  The cost of the intervention and implementation can influence the adoption and sustainability; interventions maybe more difficult to sustain if they were supported as part of a research study. | Costs are a central motivating reason for the interventions of handwashing and bleach themselves, and the study mentions simply that the weekly visits would be prohibitively expensive at scale. | Fair |
| **Criterion #6** |  |  |
| **Criterion #7**  **Population needs**  (From CFIR, Damschroder, 2009)  **Explanation/Example:**  The extent to which population needs, as well as barriers and facilitators to meet those needs, are accurately known and prioritized. This could include population-based data on causes of morbidity and mortality, political or cultural barriers or facilitators, and/or more locally focused data about local needs, barriers or facilitators. | “In these communities, infant mortality is high, and 40% of all deaths among children less than five years of age are due to diarrhea. In prior studies, households in these communities that added dilute bleach to their highly contaminated drinking water and stored it in vessels that prevented recontamination had markedly less contaminated water than households with standard water handling practices.” | Good |
| **Criterion #8**  **Process of implementation:** Description of facilitators or barriers which have influenced the intervention or program’s implementation (see #10) revealed by a process assessment.  In contrast to the criterion #7 above which assesses barriers and facilitators as inputs to developing the intervention strategy, this criterion assesses the actual barriers and facilitators identified during and after the implementation.  (From CReDECI, Mohler, 2012; also mentioned in Michie, 2009)  **Explanation/Example:**  "The attitudes of the nursing home managers turned out to be an important factor supporting or impeding the success of the intervention's implementation. The more the managers agreed with the interventions’ aim, the better the nursing staff felt supported." | No text found. | Poor/none |
| **Criterion #9**  **Description of materials:** Description of all materials or tools used for the implementation  (From CReDECI, Mohler, 2012)  **Explanation/Example:**  "The primary enablers of behaviour change were paid community-based health workers, who were recruited from the local community based on 12 years or more of education,  proficient communication and reasoning skills, commitment towards community work, and references of community stakeholders. They received a combination of classroombased  and apprentice ship-based field training over 7 days on knowledge, attitudes, and practices related to essential newborn care within the community, behaviour change management, and trust-building. After training, suitable candidates were closely mentored and supervised by a regional programme supervisor (n=4) responsible for 6–7 trainees, for an additional week before final selection was made." | There is only passing reference to “encouragement” given to households to reinforce the importance of water treatment without further detail on these components. Also, there is no further description of any of the pamphlets, videos, etc. There is this description of the implementers: “HOPE field workers who have completed at least 8 years of education and represented a variety of ethnic and linguistic groups and lived either in the study communities or nearby communities.” | Poor |
| **Criterion #10**  **Process of Implementation:** Description of an assessment of the implementation process  (From CReDECI, Mohler 2012)  **Explanation/Example:**  Process assessment is a prerequisite for determining the success of the intervention's implementation and should be an integral part of an assessment of the intervention’s effect. For example, "To gain insight into the dissemination and the delivery of the  intervention and to draw conclusions about potential barriers and facilitators to implementing the intervention in other settings, data on the implementation process were collected alongside the randomized-controlled trial. Therefore, we assessed the quality of  delivery of the interventional components (observed by members of the research team not involved in the delivery of the intervention) and the adherence to study protocol (number  and type of deviations from the protocol, using a pilot-tested standardized form). We also analyzed barriers and facilitators for the delivery of intervention’s components (focus group interviews with intervention participants)." | No relevant text found, but need for further implementation in other settings to determine potential success of the intervention is mentioned. | Poor/none |

**Additional File 2-B. - Criterion Table for Lule et al.; 2005**

| **Criterion** | **Example of text related to this criterion** | **Rating** |
| --- | --- | --- |
| **Criterion #1**  **Intervention Characteristics:** Intervention/Program source  (From CFIR, Damschroder, 2009)  **Explanation/Example:**  Is the intervention/program externally or internally developed? An intervention/program may be internally developed as a good idea, a solution to a problem, or other grass roots effort, or may be developed by an external entity (such as a foundation or a NGO). Interventions or programs that arise internally from the populations who will be impacted are sometimes more sustainable than externally developed programs dependent on external funding. The perceived legitimacy of the source may also influence implementation. | The Safe Water System (SWS), a household-based water quality intervention developed by the Centers for Disease Control and Prevention (CDC) and the Pan American Health Organization, consists of water treatment using locally produced sodium hypochlorite solution and safe water storage in a narrow-mouth container.(It was externally developed) | Good |
| **Criterion #2**  **Intervention Characteristics:** A description of why the intervention was hypothesized to have an impact on the outcome, according to theory. (From CReDECI, Mohler 2012; also mentioned in Michie, 2009)  **Explanation/Example:**  The theoretical basis of the intervention should be clearly stated. This includes the theory on which the intervention is founded as well as, if available, empirical evidence from studies in different settings or countries. For example, "The implementation was based on Rogers’ Diffusion of Innovation theory, which posits 5 factors of innovation that influence a decision to adopt or reject an innovation: relative advantage, compatibility, complexity or simplicity, trialability, observability. A similar intervention, also based on Rogers’ Diffusion of Innovation theory, was successfully implemented in other countries." | No text found. | Poor/none |
| **Criterion #3**  **Intervention Characteristics:**  Rationale for the aim/essential functions of the intervention/program’s components, including the evidence whether the components are appropriate for achieving this goal.  This differs from the need to articulate the theory behind the intervention in that the theory posits the general principles (such as Rogers Diffusion of Innovation) while this item is about specific components of the intervention and the effects of the component on specific targets.  (From CReDECI, Mohler, 2012; also mentioned in Michie, 2009) | No text found. | Poor/none |
| **Criterion #4**  **Intervention Characteristics:**  Detailed description of the intervention/program (From WIDER as described in Michie, 2009)  **The detailed description should include:**  a. Characteristics of those delivering the intervention/program (such as a nurse or lay health worker)  b. Characteristics of the recipients  c. The setting  d. The mode of delivery (such as face-to-face)  e. The intensity of the intervention/program (such as the contact time with participants)  f. The duration (such as the number of sessions and their spacing interval over a given period)  g. Adherence or fidelity to delivery protocols  h. A detailed description of the intervention/program content provided to each study group | a. “study staff visited participants homes”  b. The population’s HIV status and water handling practices are described in tables 1 and 2, along with age. But that is all (e.g., no education, assets, etc.).  c. The setting is patients of a rural HIV clinic and their families in Uganda without access to chlorinated municipal water.  d. The intervention is delivered face-to-face. “Study staff visited participants’ homes to conduct a census and obtain consent from household members….”  e. The study design section mentions weekly visits, but not how long these lasted for. It also mentions lasting for 5 months, but then mentions data on diarrheal incidence were collected until November 2002 (having started in January 2001).  f. fair (see comments in e)  g. No mention of this by those tasked with implementing the intervention.  h. “Field workers educated participants in the intervention group how to use the SWS< and replenished the solution as needed during weekly visits. To minimize confounding by exposure to health education messages, field workers instructed both intervention and control households on hygiene and education.” | Poor  Fair  Fair  Good  Fair  Fair  Poor  Poor |
| **Criterion #5**  **Intervention Characteristics:**  Costs of the intervention and costs associated with implementing the intervention (From CFIR, Damschroder, 2009; CReDECI, Mohler, 2012)  **Explanation/Example:**  The cost of the intervention and implementation can influence the adoption and sustainability; interventions maybe more difficult to sustain if they were supported as part of a research study. | No mention of costs. | Poor |
| **Criterion #6** |  |  |
| **Criterion #7**  **Population needs**  (From CFIR, Damschroder, 2009)  **Explanation/Example:**  The extent to which population needs, as well as barriers and facilitators to meet those needs, are accurately known and prioritized. This could include population-based data on causes of morbidity and mortality, political or cultural barriers or facilitators, and/or more locally focused data about local needs, barriers or facilitators. | The motivation for the study is that people living with HIV in low resource settings often fall victim to opportunistic infections such as diarrhea, whether or not there’s ARV available. But nothing specifically local to this area outside of having an HIV clinic and high prevalence (along with unsafe water). | Fair |
| **Criterion #8**  **Process of implementation:** Description of facilitators or barriers which have influenced the intervention or program’s implementation (see #10) revealed by a process assessment.  In contrast to the criterion #7 above which assesses barriers and facilitators as inputs to developing the intervention strategy, this criterion assesses the actual barriers and facilitators identified during and after the implementation.  (From CReDECI, Mohler, 2012; also mentioned in Michie, 2009)  **Explanation/Example:**  "The attitudes of the nursing home managers turned out to be an important factor supporting or impeding the success of the intervention's implementation. The more the managers agreed with the interventions’ aim, the better the nursing staff felt supported." | No text found | Poor/none |
| **Criterion #9**  **Description of materials:** Description of all materials or tools used for the implementation  (From CReDECI, Mohler, 2012)  **Explanation/Example:**  "The primary enablers of behaviour change were paid community-based health workers, who were recruited from the local community based on 12 years or more of education,  proficient communication and reasoning skills, commitment towards community work, and references of community stakeholders. They received a combination of classroombased  and apprentice ship-based field training over 7 days on knowledge, attitudes, and practices related to essential newborn care within the community, behaviour change management, and trust-building. After training, suitable candidates were closely mentored and supervised by a regional programme supervisor (n=4) responsible for 6–7 trainees, for an additional week before final selection was made." | No details outside of lengthy description of the chlorine solution and water vessel itself. | Poor |
| **Criterion #10**  **Process of Implementation:** Description of an assessment of the implementation process  (From CReDECI, Mohler 2012)  **Explanation/Example:**  Process assessment is a prerequisite for determining the success of the intervention's implementation and should be an integral part of an assessment of the intervention’s effect. For example, "To gain insight into the dissemination and the delivery of the  intervention and to draw conclusions about potential barriers and facilitators to implementing the intervention in other settings, data on the implementation process were collected alongside the randomized-controlled trial. Therefore, we assessed the quality of  delivery of the interventional components (observed by members of the research team not involved in the delivery of the intervention) and the adherence to study protocol (number  and type of deviations from the protocol, using a pilot-tested standardized form). We also analyzed barriers and facilitators for the delivery of intervention’s components (focus  group interviews with intervention participants)." | No relevant text found. | Poor/none |

**Additional File 2-C. - Criterion Table for Sobsey et al.; 2003**

| **Criterion** | **Example of text related to this criterion** | **Rating** |
| --- | --- | --- |
| **Criterion #1**  **Intervention Characteristics:** Intervention/Program source  (From CFIR, Damschroder, 2009)  **Explanation/Example:**  Is the intervention/program externally or internally developed? An intervention/program may be internally developed as a good idea, a solution to a problem, or other grass roots effort, or may be developed by an external entity (such as a foundation or a NGO). Interventions or programs that arise internally from the populations who will be impacted are sometimes more sustainable than externally developed programs dependent on external funding. The perceived legitimacy of the source may also influence implementation. | Several simple, accessible, appropriate and affordable systems to improve the microbiological quality of collected household water by treatment and to protect it by storage in an appropriate container have been identified, evaluated and are now being implemented worldwide. One is manual chlorination and storage in a narrow-mouth plastic vessel designed to minimize further contamination during storage and use (Mintz *et al*., 1995, 2001; Reiff *et al*., 1995; Quick *et al*., 1996).*.(It was externally developed)* | Good |
| **Criterion #2**  **Intervention Characteristics:** A description of why the intervention was hypothesized to have an impact on the outcome, according to theory. (From CReDECI, Mohler 2012; also mentioned in Michie, 2009)  **Explanation/Example:**  The theoretical basis of the intervention should be clearly stated. This includes the theory on which the intervention is founded as well as, if available, empirical evidence from studies in different settings or countries. For example, "The implementation was based on Rogers’ Diffusion of Innovation theory, which posits 5 factors of innovation that influence a decision to adopt or reject an innovation: relative advantage, compatibility, complexity or simplicity, trialability, observability. A similar intervention, also based on Rogers’ Diffusion of Innovation theory, was successfully implemented in other countries." | No relevant text was found. | Poor/none |
| **Criterion #3**  **Intervention Characteristics:**  Rationale for the aim/essential functions of the intervention/program’s components, including the evidence whether the components are appropriate for achieving this goal.  This differs from the need to articulate the theory behind the intervention in that the theory posits the general principles (such as Rogers Diffusion of Innovation) while this item is about specific components of the intervention and the effects of the component on specific targets.  (From CReDECI, Mohler, 2012; also mentioned in Michie, 2009) | No relevant text was found. | Poor/none |
| **Criterion #4**  **Intervention Characteristics:**  Detailed description of the intervention/program (From WIDER as described in Michie, 2009)  **The detailed description should include:**  a. Characteristics of those delivering the intervention/program (such as a nurse or lay health worker)  b. Characteristics of the recipients  c. The setting  d. The mode of delivery (such as face-to-face)  e. The intensity of the intervention/program (such as the contact time with participants)  f. The duration (such as the number of sessions and their spacing interval over a given period)  g. Adherence or fidelity to delivery protocols  h. A detailed description of the intervention/program content provided to each study group | a. “trained health workers”  b. “One study site consisted of two peri-urban settlements near a city of 70,000 people in subtropical Bolivia where shallow groundwater was collected for use by households. The other study was in an informal urban settlement in Dhaka City, Bangladesh, where households collected water primarily from clandestine connections to the municipal water supply which provided water of variable quality and only for a few hours per day. Both study sites had populations of low socioeconomic status and poor sanitation conditions (no sewerage systems and only some latrines).”  c. Identical text as pasted in 5B.  d. The intervention is delivered face-to-face. “Study housheolds were visited weekly by trained health workers who obtained information about household cases of diarrhea….”  e. In Bolivia all households were given health education at the start, but no further details were provided.  f. The study design section mentions weekly visits, but not how long these lasted for. It also mentions lasting for 8 months in Bangladesh and 6 months in Bolivia.  g. No mention of this by those tasked with implementing the intervention.  h. “In both studies intervention households were given basic instruction on the use of the intervention. In Bolivia intervention and control households additionally received health education in hygiene and sanitation measures to prevent diarrheal disease.” | Poor  Poor  Good  Good  Poor  Good  Poor  Poor |
| **Criterion #5**  **Intervention Characteristics:**  Costs of the intervention and costs associated with implementing the intervention (From CFIR, Damschroder, 2009; CReDECI, Mohler, 2012)  **Explanation/Example:**  The cost of the intervention and implementation can influence the adoption and sustainability; interventions maybe more difficult to sustain if they were supported as part of a research study. | No mention of costs, but says it’s a cost-effective intervention (without comment on weekly visits their study included). | Poor |
| **Criterion #6** |  |  |
| **Criterion #7**  **Population needs**  (From CFIR, Damschroder, 2009)  **Explanation/Example:**  The extent to which population needs, as well as barriers and facilitators to meet those needs, are accurately known and prioritized. This could include population-based data on causes of morbidity and mortality, political or cultural barriers or facilitators, and/or more locally focused data about local needs, barriers or facilitators. | The motivation for the study is again pretty clear that waterborne illnesses pose huge burden of disease and many places lack piped and treated water supplies. | Fair |
| **Criterion #8**  **Process of implementation:** Description of facilitators or barriers which have influenced the intervention or program’s implementation (see #10) revealed by a process assessment.  In contrast to the criterion #7 above which assesses barriers and facilitators as inputs to developing the intervention strategy, this criterion assesses the actual barriers and facilitators identified during and after the implementation.  (From CReDECI, Mohler, 2012; also mentioned in Michie, 2009)  **Explanation/Example:**  "The attitudes of the nursing home managers turned out to be an important factor supporting or impeding the success of the intervention's implementation. The more the managers agreed with the interventions’ aim, the better the nursing staff felt supported." | “The system was widely used, accepted and considered affordable by the participating communities based on compliance, acceptability and willingness to pay studies (data not shown) (Venczel, 1997; Handzel, 1998).” | Fair |
| **Criterion #9**  **Description of materials:** Description of all materials or tools used for the implementation  (From CReDECI, Mohler, 2012)  **Explanation/Example:**  "The primary enablers of behaviour change were paid community-based health workers, who were recruited from the local community based on 12 years or more of education,  proficient communication and reasoning skills, commitment towards community work, and references of community stakeholders. They received a combination of classroombased  and apprentice ship-based field training over 7 days on knowledge, attitudes, and practices related to essential newborn care within the community, behaviour change management, and trust-building. After training, suitable candidates were closely mentored and supervised by a regional programme supervisor (n=4) responsible for 6–7 trainees, for an additional week before final selection was made." | No details outside of lengthy description of the chlorine solution and water vessel itself. | Poor |
| **Criterion #10**  **Process of Implementation:** Description of an assessment of the implementation process  (From CReDECI, Mohler 2012)  **Explanation/Example:**  Process assessment is a prerequisite for determining the success of the intervention's implementation and should be an integral part of an assessment of the intervention’s effect. For example, "To gain insight into the dissemination and the delivery of the  intervention and to draw conclusions about potential barriers and facilitators to implementing the intervention in other settings, data on the implementation process were collected alongside the randomized-controlled trial. Therefore, we assessed the quality of  delivery of the interventional components (observed by members of the research team not involved in the delivery of the intervention) and the adherence to study protocol (number  and type of deviations from the protocol, using a pilot-tested standardized form). We also analyzed barriers and facilitators for the delivery of intervention’s components (focus  group interviews with intervention participants)." | No relevant text found. | Poor/none |

**Preventing Mother-to-Child Transmission**

**Additional File 2-D. Criterion Table for Futterman et al.; 2010**

| **Criterion** | **Example of text related to this criterion** | **Rating** |
| --- | --- | --- |
| **Criterion #1**  **Intervention Characteristics:** Intervention/Program source  (From CFIR, Damschroder, 2009)  **Explanation/Example:**  Is the intervention/program externally or internally developed? An intervention/program may be internally developed as a good idea, a solution to a problem, or other grass roots effort, or may be developed by an external entity (such as a foundation or a NGO). Interventions or programs that arise internally from the populations who will be impacted are sometimes more sustainable than externally developed programs dependent on external funding. The perceived legitimacy of the source may also influence implementation. | Supported in part by the Center for AIDS Research at the Albert Einstein College of Medicine, funded by the US National Institute of Health (NIH) and the National Institute for Allergy and Infectious Disease (NIAID).  The mothers2mothers (M2M) program currently operates in over 500 communities in 7 African nations. | Fair |
| **Criterion #2**  **Intervention Characteristics:** A description of why the intervention was hypothesized to have an impact on the outcome, according to theory. (From CReDECI, Mohler 2012; also mentioned in Michie, 2009)  **Explanation/Example:**  The theoretical basis of the intervention should be clearly stated. This includes the theory on which the intervention is founded as well as, if available, empirical evidence from studies in different settings or countries. For example, "The implementation was based on Rogers’ Diffusion of Innovation theory, which posits 5 factors of innovation that influence a decision to adopt or reject an innovation: relative advantage, compatibility, complexity or simplicity, trial ability, observability. A similar intervention, also based on Rogers’ Diffusion of Innovation theory, was successfully implemented in other countries." | Cognitive–behavioral interventions that were developed for people living with HIV in the USA have improved health behaviors and reduced mental health symptoms. | Good |
| **Criterion #3**  **Intervention Characteristics:**  Rationale for the aim/essential functions of the intervention/program’s components, including the evidence whether the components are appropriate for achieving this goal.  This differs from the need to articulate the theory behind the intervention in that the theory posits the general principles (such as Rogers Diffusion of Innovation) while this item is about specific components of the intervention and the effects of the component on specific targets.  (From CReDECI, Mohler, 2012; also mentioned in Michie, 2009) | The Mamekhaya (‘‘respect for women’’ in the Xhosa language) program was created to pair the strengths of CBIs developed in the USA with theM2M  model. In the first phase, described by Futterman and colleagues (2006), the elements of effective CBIs were culturally adapted to be useful in the environment of South African perinatal care for HIV-positive women. | Fair |
| **Criterion #4**  **Intervention Characteristics:**  Detailed description of the intervention/program (From WIDER as described in Michie, 2009)  **The detailed description should include:**  a. Characteristics of those delivering the intervention/program (such as a nurse or lay health worker)  b. Characteristics of the recipients  c. The setting  d. The mode of delivery (such as face-to-face)  e. The intensity of the intervention/program (such as the contact time with participants)  f. The duration (such as the number of sessions and their spacing interval over a given period)  g. Adherence or fidelity to delivery protocols  h. A detailed description of the intervention/program content provided to each study group | a. Women were linked to mentor mothers who were also HIV positive, had children recently, had used PMTCT services, and were coping positively.  b. HIV positive pregnant women living in Cape Town. Mean age 26.5 years, native language Xhosa. 36.3% were living with husband or partner, 31.9% were employed.  c. Two antenatal clinics in Cape Town, South Africa  d. Face to face  e. Women attended an 8 session small group cognitive behavioral intervention.  f. Mentor mothers provided support through pregnancy and in the weeks following delivery.  g. Only 44% of patients completed a follow-up interview. Women lost to follow-up were less likely to be married or live with their partner.  For evaluation, participants were asked about  PMTCT actions  Use of ARV during pregnancy  Providing ARVs for newborn  Testing baby for HIV  Exclusive infant-feeding methods  Post-delivery f/u visits  Whether partner had been tested  Practice of safe sex  Knowledge of HIV (14 items)  h. 8 CBI sessions focused on four broad topics: (1) Healthy Living (staying in care, dealing with symptoms, learning about HIV and when to take ARVs); (2) Feeling Happy and Strong (disclosure, dealing with stigma, finding support, feeling hope, avoiding negative emotions, dealing with domestic violence and substance abuse); (3) Partnering and Preventing Transmission (infant-feeding practices, general HIV precautions, partner testing, disclosure, safer sex, condom use, and family planning); and (4) Parenting (feeding choice, immunization of the baby, adherence to pre- and post-natal baby treatment,  testing the baby, planning custody, forming an attachment to the baby). All sessions followed the same format, with each including a review of recent  experiences; role plays; a didactic component; paired conversations related to the current discussion topic; group discussion and brainstorming; music, meditation, and breathing exercises; and goal-setting. | Good  Good  Fair  Good  Fair  Good  Good  Good |
| **Criterion #5**  **Intervention Characteristics:**  Costs of the intervention and costs associated with implementing the intervention (From CFIR, Damschroder, 2009; CReDECI, Mohler, 2012)  **Explanation/Example:**  The cost of the intervention and implementation can influence the adoption and sustainability; interventions maybe more difficult to sustain if they were supported as part of a research study. | Not described. | Poor |
| **Criterion #6**  Outer Setting: External policies and incentives (From CFIR, Damschroder, 2009)  **Explanation/Example:**  How does the health service, intervention, or program relate to country and global health goals? Is the program part of a larger strategy? If so how is it strategically aligned? A country's health policies may influence the implementation of a particular intervention or program. |  |  |
| **Criterion #7**  **Population needs**  (From CFIR, Damschroder, 2009)  **Explanation/Example:**  The extent to which population needs, as well as barriers and facilitators to meet those needs, are accurately known and prioritized. This could include population-based data on causes of morbidity and mortality, political or cultural barriers or facilitators, and/or more locally focused data about local needs, barriers or facilitators. | 28% of pregnant women attending antenatal clinics in South Africa are HIV positive. Women must face the challenges of following PMTCT programs that required practice count to community norms like breast feeding. | Good |
| **Criterion #8**  **Process of implementation:** Description of facilitators or barriers which have influenced the intervention or program’s implementation (see #10) revealed by a process assessment.  In contrast to the criterion #7 above which assesses barriers and facilitators as inputs to developing the intervention strategy, this criterion assesses the actual barriers and facilitators identified during and after the implementation.  (From CReDECI, Mohler, 2012; also mentioned in Michie, 2009)  **Explanation/Example:**  "The attitudes of the nursing home managers turned out to be an important factor supporting or impeding the success of the intervention's implementation. The more the managers agreed with the interventions’ aim, the better the nursing staff felt supported." | Only 44% of patients completed a follow-up interview. Women lost to follow-up were less likely to be married or live with their partner.  There was no statistically significant difference between control (usual care) and intervention participants in PMTCT-related actions or other transmission risk behaviors. | Fair |
| **Criterion #9**  **Description of materials:** Description of all materials or tools used for the implementation  (From CReDECI, Mohler, 2012)  **Explanation/Example:**  "The primary enablers of behavior change were paid community-based health workers, who were recruited from the local community based on 12 years or more of education,  proficient communication and reasoning skills, commitment towards community work, and references of community stakeholders. They received a combination of class room based  and apprentice ship-based field training over 7 days on knowledge, attitudes, and practices related to essential newborn care within the community, behaviour change management, and trust-building. After training, suitable candidates were closely mentored and supervised by a regional programme supervisor (n=4) responsible for 6–7 trainees, for an additional week before final selection was made." | Women attended an 8 session small group cognitive behavioral intervention. Conducted by two mentor mothers trained in CBI skills. Training included review of the intervention content, structure of the intervention modules, and pilot sessions.  All sessions followed the same format, with each including a review of recent experiences; role plays; a didactic component; paired conversation related to the current discussion topic; group discussion; brainstorming; music; meditation / breathing exercises, and goal-setting. | Fair |
| **Criterion #10**  **Process of Implementation:** Description of an assessment of the implementation process  (From CReDECI, Mohler 2012)  **Explanation/Example:**  Process assessment is a prerequisite for determining the success of the intervention's implementation and should be an integral part of an assessment of the intervention’s effect. For example, "To gain insight into the dissemination and the delivery of the  intervention and to draw conclusions about potential barriers and facilitators to implementing the intervention in other settings, data on the implementation process were collected alongside the randomized-controlled trial. Therefore, we assessed the quality of  delivery of the interventional components (observed by members of the research team not involved in the delivery of the intervention) and the adherence to study protocol (number  and type of deviations from the protocol, using a pilot-tested standardized form). We also analyzed barriers and facilitators for the delivery of intervention’s components (focus  group interviews with intervention participants)." | Women were asked about  Testing baby for HIV  Post-delivery f/u visits  Whether partner had been tested  Knowledge of HIV (14 items)  Types of support (20 items) | Fair |

**Additional File 2-E. Criterion Table for Torpey et al.; 2010**

| **Criterion** | **Example of text related to this criterion** | **Rating** |
| --- | --- | --- |
| **Criterion #1**  **Intervention Characteristics:** Intervention/Program source  (From CFIR, Damschroder, 2009)  **Explanation/Example:**  Is the intervention/program externally or internally developed? An intervention/program may be internally developed as a good idea, a solution to a problem, or other grass roots effort, or may be developed by an external entity (such as a foundation or a NGO). Interventions or programs that arise internally from the populations who will be impacted are sometimes more sustainable than externally developed programs dependent on external funding. The perceived legitimacy of the source may also influence implementation. | Funded by Elizabeth Glaser Pediatric AIDS Foundation. Evaluation funded by US National Institutes of Health (NIH) Fogarty Center. | Good |
| **Criterion #2**  **Intervention Characteristics:** A description of why the intervention was hypothesized to have an impact on the outcome, according to theory. (From CReDECI, Mohler 2012; also mentioned in Michie, 2009)  **Explanation/Example:**  The theoretical basis of the intervention should be clearly stated. This includes the theory on which the intervention is founded as well as, if available, empirical evidence from studies in different settings or countries. For example, "The implementation was based on Rogers’ Diffusion of Innovation theory, which posits 5 factors of innovation that influence a decision to adopt or reject an innovation: relative advantage, compatibility, complexity or simplicity, trialability, observability. A similar intervention, also based on Rogers’ Diffusion of Innovation theory, was successfully implemented in other countries." | The majority of pregnant women in stable relations who are tested as part of routine care do not inform their partner of positive HIV results, fearing domestic violence, abandonment, or stigmatization. We hypothesized the conducting Voluntary Counseling and Testing (VCT) for pregnant women together with partners could facilitated notification and increase partner participation in the decision making process. Studies among discordant couples in non-antenatal settings support this hypothesis. | Fair |
| **Criterion #3**  **Intervention Characteristics:**  Rationale for the aim/essential functions of the intervention/program’s components, including the evidence whether the components are appropriate for achieving this goal.  This differs from the need to articulate the theory behind the intervention in that the theory posits the general principles (such as Rogers Diffusion of Innovation) while this item is about specific components of the intervention and the effects of the component on specific targets.  (From CReDECI, Mohler, 2012; also mentioned in Michie, 2009) | The majority of pregnant women in stable relations who are tested as part of routine care do not inform their partner of positive HIV results, fearing domestic violence, abandonment, or stigmatization. We hypothesized the conducting Voluntary Counseling and Testing (VCT) for pregnant women together with partners could facilitated notification and increase partner participation in the decision making process. Studies among discordant couples in non-antenatal settings support this hypothesis. | Fair |
| **Criterion #4**  **Intervention Characteristics:**  Detailed description of the intervention/program (From WIDER as described in Michie, 2009)  **The detailed description should include:**  a. Characteristics of those delivering the intervention/program (such as a nurse or lay health worker)  b. Characteristics of the recipients  c. The setting  d. The mode of delivery (such as face-to-face)  e. The intensity of the intervention/program (such as the contact time with participants)  f. The duration (such as the number of sessions and their spacing interval over a given period)  g. Adherence or fidelity to delivery protocols  h. A detailed description of the intervention/program content provided to each study group | a. Women and men were counseled by a female or male HIV infected peer counselor, respectively.  b. Pregnant women; 54% were married, 90% lived with partner or spouse, 29% were employed.  c. Public antenatal clinic in Nairobi, Kenya  d. Face to face  e. Enrolled at first antenatal visit; counseling in group. Women asked to return within 7 days For lab tests and optional VCT with rapid results. Additional counseling 2 weeks later.  f. Follow-up 1 week after birth, and 3 & 6 months postpartum.  g. 2836 women enrolled in the study (Fig. 1). A total of 2231 (79%)  returned to the clinic for a second study visit, of whom 2104 (94%) accepted HIV-1 counseling and testing and 1991 (89%) reported inviting their partner for HIV-1 counseling. Among 308 partners who came for testing, 116 couples (38%) elected to receive posttest counseling together and 192 (62%) to receive posttest counseling individually (Fig. 1). A total of 170 (55%) of the 308 men who presented for the first VCT visit returned for additional counseling on interventions to prevent mother-to-child transmission approximately 2 weeks after receiving HIV-1 test results.  h. Described at length. Example: Women were provided information as a group about heterosexual and mother to child HIV transmission and encouraged to inform male partners of VCT availability. The pros and cons of partner testing, couples VCT, and partner notification of HIV tests results were discussed on an individual basis. | Fair  Good  Fair  Good  Fair  Good  Good  Good |
| **Criterion #5**  **Intervention Characteristics:**  Costs of the intervention and costs associated with implementing the intervention (From CFIR, Damschroder, 2009; CReDECI, Mohler, 2012)  **Explanation/Example:**  The cost of the intervention and implementation can influence the adoption and sustainability; interventions maybe more difficult to sustain if they were supported as part of a research study. | Not described. | Poor |
| **Criterion #6**  **Outer Setting:** External policies and incentives (From CFIR, Damschroder, 2009)  **Explanation/Example:**  How does the health service, intervention, or program relate to country and global health goals? Is the program part of a larger strategy? If so how is it strategically aligned? A country's health policies may influence the implementation of a particular intervention or program. |  |  |
| **Criterion #7**  **Population needs**  (From CFIR, Damschroder, 2009)  **Explanation/Example:**  The extent to which population needs, as well as barriers and facilitators to meet those needs, are accurately known and prioritized. This could include population-based data on causes of morbidity and mortality, political or cultural barriers or facilitators, and/or more locally focused data about local needs, barriers or facilitators. | In both research and non-research settings in sub-Saharan Africa, <75% of HIV infected pregnant women who are tested learn their HIV status and <50% of these obtain antiretrovirals or use condoms postpartum. | Fair |
| **Criterion #8**  **Process of implementation:** Description of facilitators or barriers which have influenced the intervention or program’s implementation (see #10) revealed by a process assessment.  In contrast to the criterion #7 above which assesses barriers and facilitators as inputs to developing the intervention strategy, this criterion assesses the actual barriers and facilitators identified during and after the implementation.  (From CReDECI, Mohler, 2012; also mentioned in Michie, 2009)  **Explanation/Example:**  "The attitudes of the nursing home managers turned out to be an important factor supporting or impeding the success of the intervention's implementation. The more the managers agreed with the interventions’ aim, the better the nursing staff felt supported." | A lack of community awareness about the importance of partner VCT and cultural beliefs that men should not participate in antenatal activities led to low partner involvement.  Poor uptake of VCT and couple counseling may also have been due to women not informing partners of VCT availability or conflicts between clinic hours and men’s work schedules. | Good |
| **Criterion #9**  **Description of materials:** Description of all materials or tools used for the implementation  (From CReDECI, Mohler, 2012)  **Explanation/Example:**  "The primary enablers of behaviour change were paid community-based health workers, who were recruited from the local community based on 12 years or more of education,  proficient communication and reasoning skills, commitment towards community work, and references of community stakeholders. They received a combination of class room based  and apprentice ship-based field training over 7 days on knowledge, attitudes, and practices related to essential newborn care within the community, behaviour change management, and trust-building. After training, suitable candidates were closely mentored and supervised by a regional programme supervisor (n=4) responsible for 6–7 trainees, for an additional week before final selection was made." | Well described; for example: In addition to counseling….rapid assays were performed for HIV-1, syphilis, and hemoglobin and results were available within 30 minutes. Each women and man was asked confidentially whether she or he would prefer to receive test results and post-test counseling alone or as a couple. During the counseling session…condoms were offered to all in conjunction with counseling on safe sex and breast feeding. | Good |
| **Criterion #10**  **Process of Implementation:** Description of an assessment of the implementation process  (From CReDECI, Mohler 2012)  **Explanation/Example:**  Process assessment is a prerequisite for determining the success of the intervention's implementation and should be an integral part of an assessment of the intervention’s effect. For example, "To gain insight into the dissemination and the delivery of the  intervention and to draw conclusions about potential barriers and facilitators to implementing the intervention in other settings, data on the implementation process were collected alongside the randomized-controlled trial. Therefore, we assessed the quality of  delivery of the interventional components (observed by members of the research team not involved in the delivery of the intervention) and the adherence to study protocol (number  and type of deviations from the protocol, using a pilot-tested standardized form). We also analyzed barriers and facilitators for the delivery of intervention’s components (focus  group interviews with intervention participants)." | Correlates of couple counseling, partner presentation for VCT, and partner notification were determined. Nevirapine uptake and infant feeding choices were evaluated with respect to partner involvement, couple counseling, and partner notification of HIV status. | Fair |

**Additional File 2-F. Criterion Table for Farquhar et al.; 2004**

| **Criterion** | **Example of text related to this criterion** | **Rating** |
| --- | --- | --- |
| **Criterion #1**  **Intervention Characteristics:** Intervention/Program source  (From CFIR, Damschroder, 2009)  **Explanation/Example:**  Is the intervention/program externally or internally developed? An intervention/program may be internally developed as a good idea, a solution to a problem, or other grass roots effort, or may be developed by an external entity (such as a foundation or a NGO). Interventions or programs that arise internally from the populations who will be impacted are sometimes more sustainable than externally developed programs dependent on external funding. The perceived legitimacy of the source may also influence implementation. | Funded by Elizabeth Glaser Pediatric AIDS Foundation. Evaluation funded by US National Institutes of Health (NIH) Fogarty Center. | Good |
| **Criterion #2**  **Intervention Characteristics:** A description of why the intervention was hypothesized to have an impact on the outcome, according to theory. (From CReDECI, Mohler 2012; also mentioned in Michie, 2009)  **Explanation/Example:**  The theoretical basis of the intervention should be clearly stated. This includes the theory on which the intervention is founded as well as, if available, empirical evidence from studies in different settings or countries. For example, "The implementation was based on Rogers’ Diffusion of Innovation theory, which posits 5 factors of innovation that influence a decision to adopt or reject an innovation: relative advantage, compatibility, complexity or simplicity, trialability, observability. A similar intervention, also based on Rogers’ Diffusion of Innovation theory, was successfully implemented in other countries." | The majority of pregnant women in stable relations who are tested as part of routine care do not inform their partner of positive HIV results, fearing domestic violence, abandonment, or stigmatization. We hypothesized the conducting Voluntary Counseling and Testing (VCT) for pregnant women together with partners could facilitated notification and increase partner participation in the decision making process. Studies among discordant couples in non-antenatal settings support this hypothesis. | Fair |
| **Criterion #3**  **Intervention Characteristics:**  Rationale for the aim/essential functions of the intervention/program’s components, including the evidence whether the components are appropriate for achieving this goal.  This differs from the need to articulate the theory behind the intervention in that the theory posits the general principles (such as Rogers Diffusion of Innovation) while this item is about specific components of the intervention and the effects of the component on specific targets.  (From CReDECI, Mohler, 2012; also mentioned in Michie, 2009) | The majority of pregnant women in stable relations who are tested as part of routine care do not inform their partner of positive HIV results, fearing domestic violence, abandonment, or stigmatization. We hypothesized the conducting Voluntary Counseling and Testing (VCT) for pregnant women together with partners could facilitated notification and increase partner participation in the decision making process. Studies among discordant couples in non-antenatal settings support this hypothesis. | Fair |
| **Criterion #4**  **Intervention Characteristics:**  Detailed description of the intervention/program (From WIDER as described in Michie, 2009)  **The detailed description should include:**  a. Characteristics of those delivering the intervention/program (such as a nurse or lay health worker)  b. Characteristics of the recipients  c. The setting  d. The mode of delivery (such as face-to-face)  e. The intensity of the intervention/program (such as the contact time with participants)  f. The duration (such as the number of sessions and their spacing interval over a given period)  g. Adherence or fidelity to delivery protocols  h. A detailed description of the intervention/program content provided to each study group | a. Women and men were counseled by a female or male HIV infected peer counselor, respectively.  b. Pregnant women; 54% were married, 90% lived with partner or spouse, 29% were employed.  c. Public antenatal clinic in Nairobi, Kenya  d. Face to face  e. Enrolled at first antenatal visit; counseling in group. Women asked to return within 7 days For lab tests and optional VCT with rapid results. Additional counseling 2 weeks later.  f. Follow-up 1 week after birth, and 3 & 6 months postpartum.  g. 2836 women enrolled in the study (Fig. 1). A total of 2231 (79%)  returned to the clinic for a second study visit, of whom 2104 (94%) accepted HIV-1 counseling and testing and 1991 (89%) reported inviting their partner for HIV-1 counseling. Among 308 partners who came for testing, 116 couples (38%) elected to receive posttest counseling together and 192 (62%) to receive posttest counseling individually (Fig. 1). A total of 170 (55%) of the 308 men who presented for the first VCT visit returned for additional counseling on interventions to prevent mother-to-child transmission approximately 2 weeks after receiving HIV-1 test results.  h. Described at length. Example: Women were provided information as a group about heterosexual and mother to child HIV transmission and encouraged to inform male partners of VCT availability. The pros and cons of partner testing, couples VCT, and partner notification of HIV tests results were discussed on an individual basis. | Fair  Good  Fair  Good  Fair  Good  Good  Good |
| **Criterion #5**  **Intervention Characteristics:**  Costs of the intervention and costs associated with implementing the intervention (From CFIR, Damschroder, 2009; CReDECI, Mohler, 2012)  **Explanation/Example:**  The cost of the intervention and implementation can influence the adoption and sustainability; interventions maybe more difficult to sustain if they were supported as part of a research study. | Not described. | Poor |
| **Criterion #6**  **Outer Setting:** External policies and incentives (From CFIR, Damschroder, 2009)  **Explanation/Example:**  How does the health service, intervention, or program relate to country and global health goals? Is the program part of a larger strategy? If so how is it strategically aligned? A country's health policies may influence the implementation of a particular intervention or program. |  |  |
| **Criterion #7**  **Population needs**  (From CFIR, Damschroder, 2009)  **Explanation/Example:**  The extent to which population needs, as well as barriers and facilitators to meet those needs, are accurately known and prioritized. This could include population-based data on causes of morbidity and mortality, political or cultural barriers or facilitators, and/or more locally focused data about local needs, barriers or facilitators. | In both research and non-research settings in sub-Saharan Africa, <75% of HIV infected pregnant women who are tested learn their HIV status and <50% of these obtain antiretrovirals or use condoms postpartum. | Fair |
| **Criterion #8**  **Process of implementation:** Description of facilitators or barriers which have influenced the intervention or program’s implementation (see #10) revealed by a process assessment.  In contrast to the criterion #7 above which assesses barriers and facilitators as inputs to developing the intervention strategy, this criterion assesses the actual barriers and facilitators identified during and after the implementation.  (From CReDECI, Mohler, 2012; also mentioned in Michie, 2009)  **Explanation/Example:**  "The attitudes of the nursing home managers turned out to be an important factor supporting or impeding the success of the intervention's implementation. The more the managers agreed with the interventions’ aim, the better the nursing staff felt supported." | A lack of community awareness about the importance of partner VCT and cultural beliefs that men should not participate in antenatal activities led to low partner involvement.  Poor uptake of VCT and couple counseling may also have been due to women not informing partners of VCT availability or conflicts between clinic hours and men’s work schedules. | Good |
| **Criterion #9**  **Description of materials:** Description of all materials or tools used for the implementation  (From CReDECI, Mohler, 2012)  **Explanation/Example:**  "The primary enablers of behaviour change were paid community-based health workers, who were recruited from the local community based on 12 years or more of education,  proficient communication and reasoning skills, commitment towards community work, and references of community stakeholders. They received a combination of class room based  and apprentice ship-based field training over 7 days on knowledge, attitudes, and practices related to essential newborn care within the community, behaviour change management, and trust-building. After training, suitable candidates were closely mentored and supervised by a regional programme supervisor (n=4) responsible for 6–7 trainees, for an additional week before final selection was made." | Well described; for example: In addition to counseling….rapid assays were performed for HIV-1, syphilis, and hemoglobin and results were available within 30 minutes. Each women and man was asked confidentially whether she or he would prefer to receive test results and post-test counseling alone or as a couple. During the counseling session…condoms were offered to all in conjunction with counseling on safe sex and breast feeding. | Good |
| **Criterion #10**  **Process of Implementation:** Description of an assessment of the implementation process  (From CReDECI, Mohler 2012)  **Explanation/Example:**  Process assessment is a prerequisite for determining the success of the intervention's implementation and should be an integral part of an assessment of the intervention’s effect. For example, "To gain insight into the dissemination and the delivery of the  intervention and to draw conclusions about potential barriers and facilitators to implementing the intervention in other settings, data on the implementation process were collected alongside the randomized-controlled trial. Therefore, we assessed the quality of  delivery of the interventional components (observed by members of the research team not involved in the delivery of the intervention) and the adherence to study protocol (number  and type of deviations from the protocol, using a pilot-tested standardized form). We also analyzed barriers and facilitators for the delivery of intervention’s components (focus  group interviews with intervention participants)." | Correlates of couple counseling, partner presentation for VCT, and partner notification were determined. Nevirapine uptake and infant feeding choices were evaluated with respect to partner involvement, couple counseling, and partner notification of HIV status. | Fair |

**Additional File 2-G. Criterion Table for Chandisarewa et al.; 2007**

| **Criterion** | **Example of text related to this criterion** | **Rating** |
| --- | --- | --- |
| **Criterion #1**  **Intervention Characteristics:** Intervention/Program source  (From CFIR, Damschroder, 2009)  **Explanation/Example:**  Is the intervention/program externally or internally developed? An intervention/program may be internally developed as a good idea, a solution to a problem, or other grass roots effort, or may be developed by an external entity (such as a foundation or a NGO). Interventions or programs that arise internally from the populations who will be impacted are sometimes more sustainable than externally developed programs dependent on external funding. The perceived legitimacy of the source may also influence implementation. | This project was funded by the Elizabeth Glaser Pediatric AIDS Foundation and United States Agency for International Development. The Call-to-Action Project was approved by Zimbabwe Ministry of Health and Child Welfare, the Chitungwiza Health Department. | Good |
| **Criterion #2**  **Intervention Characteristics:** A description of why the intervention was hypothesized to have an impact on the outcome, according to theory. (From CReDECI, Mohler 2012; also mentioned in Michie, 2009)  **Explanation/Example:**  The theoretical basis of the intervention should be clearly stated. This includes the theory on which the intervention is founded as well as, if available, empirical evidence from studies in different settings or countries. For example, "The implementation was based on Rogers’ Diffusion of Innovation theory, which posits 5 factors of innovation that influence a decision to adopt or reject an innovation: relative advantage, compatibility, complexity or simplicity, trialability, observability. A similar intervention, also based on Rogers’ Diffusion of Innovation theory, was successfully implemented in other countries." | Provider-initiated routine antenatal HIV testing (i.e. an “opt-out” approach)  is the standard of care in the US and other developed nations. Routine antenatal HIV testing policy is rare in sub- Saharan Africa. Recent data from the PMTCT programme in Botswana demonstrated that routine HIV testing led to a significant increase in HIV test acceptance at ANC clinics. | Fair |
| **Criterion #3**  **Intervention Characteristics:**  Rationale for the aim/essential functions of the intervention/program’s components, including the evidence whether the components are appropriate for achieving this goal.  This differs from the need to articulate the theory behind the intervention in that the theory posits the general principles (such as Rogers Diffusion of Innovation) while this item is about specific components of the intervention and the effects of the component on specific targets.  (From CReDECI, Mohler, 2012; also mentioned in Michie, 2009) | PMTCT clinic counsellors held 15-minute group education and discussion  sessions with pregnant women, using a structured flip chart as a discussion  guide. The discussion focused on HIV transmission, PMTCT, sdNVP prophylaxis and routine HIV testing for all mothers, specifying the right to refuse.  Women who did not want any one of the routine antenatal tests were referred  for individual pre-test counselling to discuss their concerns. Women who arrived  for ANC when no group was conducted received the same education individually via pre-test counselling. Women who did not refuse and gave verbal informed consent individually had blood drawn for rapid HIV testing on-site  by clinic nurses in addition to routine syphilis, blood group and haemoglobin  level testing. Maternal HIV status was determined on-site using two rapid tests. | Fair |
| **Criterion #4**  **Intervention Characteristics:**  Detailed description of the intervention/program (From WIDER as described in Michie, 2009)  **The detailed description should include:**  a. Characteristics of those delivering the intervention/program (such as a nurse or lay health worker)  b. Characteristics of the recipients  c. The setting  d. The mode of delivery (such as face-to-face)  e. The intensity of the intervention/program (such as the contact time with participants)  f. The duration (such as the number of sessions and their spacing interval over a given period)  g. Adherence or fidelity to delivery protocols  h. A detailed description of the intervention/program content provided to each study group | a. Counselors are people living with HIV/AIDS who have participated in previous PMTCT clinical trials at our site.  b. Pregnant women – demographics presented in Table 2  c. Four antenatal clinics in Chitungwiza, a socio-economically  disadvantaged community 25 km south of Harare.  d. Face to face  e. 15 minute group counseling, individual counseling, HIV testing, post-test counseling, medications, follow-up by counselors. A drama skit was developed and presented at health worker in-service training workshops and at the community advisory board meetings for critiques and comments before presentation. The community counsellors performed the skit on a rotational basis at the four clinics on Tuesday, Wednesday and Thursday mornings for new ANC clients and during the afternoons in the community and at colleges, churches and industrial facilities.  f. Unclear  g. Of the 4551 pregnant women presenting for ANC during the first 6 months  of routine HIV testing, 4547 (99.9%) were tested for HIV compared with 3058 (65%) of 4700 pregnant women during the last 6 months of the opt-in testing period.  h. PMTCT clinic counselors held 15- minute group education and discussion sessions with pregnant women, using a structured flip chart as a discussion guide. The discussion focused on HIV transmission, PMTCT, sdNVP prophylaxis and routine HIV testing for all mothers, specifying the right to refuse. Women who did not want any one of the routine antenatal tests were referred for individual pre-test counseling to discuss their concerns. | Good  Good  Good  Good  Good  Poor  Good  Good |
| **Criterion #5**  **Intervention Characteristics:**  Costs of the intervention and costs associated with implementing the intervention (From CFIR, Damschroder, 2009; CReDECI, Mohler, 2012)  **Explanation/Example:**  The cost of the intervention and implementation can influence the adoption and sustainability; interventions maybe more difficult to sustain if they were supported as part of a research study. | Not described. | Poor |
| **Criterion #6**  **Outer Setting:** External policies and incentives (From CFIR, Damschroder, 2009)  **Explanation/Example:**  How does the health service, intervention, or program relate to country and global health goals? Is the program part of a larger strategy? If so how is it strategically aligned? A country's health policies may influence the implementation of a particular intervention or program. |  |  |
| **Criterion #7**  **Population needs**  (From CFIR, Damschroder, 2009)  **Explanation/Example:**  The extent to which population needs, as well as barriers and facilitators to meet those needs, are accurately known and prioritized. This could include population-based data on causes of morbidity and mortality, political or cultural barriers or facilitators, and/or more locally focused data about local needs, barriers or facilitators. | In Zimbabwe, recent estimates indicate that over 20% of women aged 15–49 years presenting for antenatal care (ANC) are HIV-infected. Transmission of HIV (PMTCT) interventions using single-dose nevirapine (sdNVP) have been implemented in many urban and rural clinics in Zimbabwe, but uptake of these interventions remains low,  Before implementation of the routine HIV testing policy, a VCT site instrument was used to assess the adequacy of staffing levels, adherence  to PMTCT protocols, availability of health education materials, availability  of test kits and medical consumables, adherence to staff roles and responsibilities, and general aspects of site operations. A counselor reflection form and a VCT client exit survey form were used to  guide the implementation of the routine HIV testing policy. | Good |
| **Criterion #8**  **Process of implementation:** Description of facilitators or barriers which have influenced the intervention or program’s implementation (see #10) revealed by a process assessment.  In contrast to the criterion #7 above which assesses barriers and facilitators as inputs to developing the intervention strategy, this criterion assesses the actual barriers and facilitators identified during and after the implementation.  (From CReDECI, Mohler, 2012; also mentioned in Michie, 2009)  **Explanation/Example:**  "The attitudes of the nursing home managers turned out to be an important factor supporting or impeding the success of the intervention's implementation. The more the managers agreed with the interventions’ aim, the better the nursing staff felt supported." | The current economic hardships in Zimbabwe and high levels of midwife  staff turnover requires more resources to be made available for training, especially to effectively communicate the new routine HIV testing approach and dispel the misconception that it is mandatory testing. | Good |
| **Criterion #9**  **Description of materials:** Description of all materials or tools used for the implementation  (From CReDECI, Mohler, 2012)  **Explanation/Example:**  "The primary enablers of behaviour change were paid community-based health workers, who were recruited from the local community based on 12 years or more of education,  proficient communication and reasoning skills, commitment towards community work, and references of community stakeholders. They received a combination of class room based  and apprentice ship-based field training over 7 days on knowledge, attitudes, and practices related to essential newborn care within the community, behaviour change management, and trust-building. After training, suitable candidates were closely mentored and supervised by a regional programme supervisor (n=4) responsible for 6–7 trainees, for an additional week before final selection was made." | PMTCT clinic counselors held 15- minute group education and discussion sessions with pregnant women, using a structured flip chart as a discussion guide. The discussion focused on HIV transmission, PMTCT, sdNVP prophylaxis and routine HIV testing for all mothers, specifying the right to refuse. Women who did not want any one of the routine antenatal tests were referred for individual pre-test counseling to discuss their concerns.  Women who did not want any one of the routine antenatal tests were referred for individual pre-test counseling to discuss their concerns. Women who arrived for ANC when no group was conducted  received the same education individually via pre-test counseling. Women  who did not refuse and gave verbal informed consent individually had blood drawn for rapid HIV testing on-site by clinic nurses in addition to routine syphilis, blood group and hemoglobin level testing.  Maternal HIV status was determined on-site using two rapid tests in  parallel (Uni-Gold Test, Trinity Biotech, USA; and Determine HIV1/2 test, Abott Laboratories, USA) on each blood sample, and a third test (OraQuick, Abott Laboratories, USA) as a tie-breaker. Women received their test results the same day during extensive individual post-test counseling, with a focus on PMTCT interventions for HIV-infected women, enrolment into support groups, counseling for exclusive breastfeeding for 6 months according to WHO and national guidelines, sdNVP prophylaxis, and mother-infant follow-up. | Good |
| **Criterion #10**  **Process of Implementation:** Description of an assessment of the implementation process  (From CReDECI, Mohler 2012)  **Explanation/Example:**  Process assessment is a prerequisite for determining the success of the intervention's implementation and should be an integral part of an assessment of the intervention’s effect. For example, "To gain insight into the dissemination and the delivery of the  intervention and to draw conclusions about potential barriers and facilitators to implementing the intervention in other settings, data on the implementation process were collected alongside the randomized-controlled trial. Therefore, we assessed the quality of  delivery of the interventional components (observed by members of the research team not involved in the delivery of the intervention) and the adherence to study protocol (number  and type of deviations from the protocol, using a pilot-tested standardized form). We also analyzed barriers and facilitators for the delivery of intervention’s components (focus  group interviews with intervention participants)." | To assess the acceptability of the routine HIV testing policy, a 15-item  self-administered exit questionnaire was administered during the initial three months of implementation to women (*n* = 2011) in Shona, the local language, after completion of their first ANC visit.  To determine if there were any negative effects related to the routine  HIV testing policy, women (*n* = 221) attending the four antenatal and postnatal clinics who had participated in routine HIV testing were interviewed individually, regardless of HIV status, during the  fifth month of study implementation. The standardized questionnaire was  administered in Shona by four trained community counselors who did not  know the client’s serostatus.  Quantitative data regarding acceptance of HIV testing and PMTCT interventions were collected according to current programme guidelines. | Good |

**Additional File 2-H. Criterion Table for Bekker et al.; 2006**

| **Criterion** | **Example of text related to this criterion** | **Rating** |
| --- | --- | --- |
| **Criterion #1**  **Intervention Characteristics:** Intervention/Program source  (From CFIR, Damschroder, 2009)  **Explanation/Example:**  Is the intervention/program externally or internally developed? An intervention/program may be internally developed as a good idea, a solution to a problem, or other grass roots effort, or may be developed by an external entity (such as a foundation or a NGO). Interventions or programs that arise internally from the populations who will be impacted are sometimes more sustainable than externally developed programs dependent on external funding. The perceived legitimacy of the source may also influence implementation. | Program was started in 2002 with funding support form an international donor, and the expansion of services in subsequent years has been supported by a grant from the Global Fund to Fight AIDS, Tuberculosis and Malaria. | Good |
| **Criterion #2**  **Intervention Characteristics:** A description of why the intervention was hypothesized to have an impact on the outcome, according to theory. (From CReDECI, Mohler 2012; also mentioned in Michie, 2009)  **Explanation/Example:**  The theoretical basis of the intervention should be clearly stated. This includes the theory on which the intervention is founded as well as, if available, empirical evidence from studies in different settings or countries. For example, "The implementation was based on Rogers’ Diffusion of Innovation theory, which posits 5 factors of innovation that influence a decision to adopt or reject an innovation: relative advantage, compatibility, complexity or simplicity, trialability, observability. A similar intervention, also based on Rogers’ Diffusion of Innovation theory, was successfully implemented in other countries." | .”…programme follows the National Ministry of Health ART guidelines, which are based on the 2002 WHO recommendations for scaling up ART in resource-poor settings.” | Fair |
| **Criterion #3**  **Intervention Characteristics:**  Rationale for the aim/essential functions of the intervention/program’s components, including the evidence whether the components are appropriate for achieving this goal.  This differs from the need to articulate the theory behind the intervention in that the theory posits the general principles (such as Rogers Diffusion of Innovation) while this item is about specific components of the intervention and the effects of the component on specific targets.  (From CReDECI, Mohler, 2012; also mentioned in Michie, 2009) | At screening visit patients were allocated to a community-based “therapeutic counselor’ living in the area….Counselors were responsible for treatment readiness group information sessions, clinic-based adherence reinforcement session and home visiting.” The counselor was the crucial factor in the intervention, but no references are given and no theory discussed. | Poor |
| **Criterion #4**  **Intervention Characteristics:**  Detailed description of the intervention/program (From WIDER as described in Michie, 2009)  **The detailed description should include:**  a. Characteristics of those delivering the intervention/program (such as a nurse or lay health worker)  b. Characteristics of the recipients  c. The setting  d. The mode of delivery (such as face-to-face)  e. The intensity of the intervention/program (such as the contact time with participants)  f. The duration (such as the number of sessions and their spacing interval over a given period)  g. Adherence or fidelity to delivery protocols  h. A detailed description of the intervention/program content provided to each study group | a. Doctors, nurses, pharmacist, and counselors.  b. Adults with AIDS diagnoses of CD4 count < 200, children with pediatric stage III disease or CD4 cell percentage < 20%.  c. Guguletu Clinic is situated in the urban Nyanga district of Cape Town, which is served by 10 primary care HIV clinics that comprised the patients’ referral base. Unemployment rate is 57%, 81% live in “informal” dwellings, HIV prevelance rate is 28% in pregnant women.  d. Face to face  e and f.  Week 0 – Screening visit  Week 2 – Blood tests  Week 4 – Tx initiation  Weeks 8, 12, 20 – Tx follow-up  16 weekly follow-ups thereafter  g. Not described  h. Patients were assessed by a doctor for symptomatic HIV-associated disease. First-line ART comprised stavudine, lamivudine plus a non-nucleoside reverse transcriptase inhibitor (efavirenz or nevirapine). The second-line regimen comprised lopinavir/ritonavir, zidovudine and didanosine. A secure supply of medication was maintained by the local health authority throughout the study period. Community-based counsellors, the majority of whom are living with HIV/AIDS, provide ongoing counseling support, addressing psychosocial issues and reinforcing the need for high levels of treatment adherence. Plasma HIV load and blood CD4 cell count were performed at week 2 and 4-monthly after commencing ART. | Good  Fair  Good  Good  e. Fair  f. Good  g. Poor  h. Fair |
| **Criterion #5**  **Intervention Characteristics:**  Costs of the intervention and costs associated with implementing the intervention (From CFIR, Damschroder, 2009; CReDECI, Mohler, 2012)  **Explanation/Example:**  The cost of the intervention and implementation can influence the adoption and sustainability; interventions maybe more difficult to sustain if they were supported as part of a research study. | Treatment was provided free to patients. Costs not reported. | Poor |
| **Criterion #6**  **Outer Setting:** External policies and incentives (From CFIR, Damschroder, 2009)  **Explanation/Example:**  How does the health service, intervention, or program relate to country and global health goals? Is the program part of a larger strategy? If so how is it strategically aligned? A country's health policies may influence the implementation of a particular intervention or program. |  |  |
| **Criterion #7**  **Population needs**  (From CFIR, Damschroder, 2009)  **Explanation/Example:**  The extent to which population needs, as well as barriers and facilitators to meet those needs, are accurately known and prioritized. This could include population-based data on causes of morbidity and mortality, political or cultural barriers or facilitators, and/or more locally focused data about local needs, barriers or facilitators. | District is socially deprived with an estimated 57% unemployment rate and with 81% of households living in informal dwellings a tuberculosis  notification rate of 1 026/100 000 and an antenatal HIV-1  seroprevalence rate of 28%, which is among the highest in the  province. | Good |
| **Criterion #8**  **Process of implementation:** Description of facilitators or barriers which have influenced the intervention or program’s implementation (see #10) revealed by a process assessment.  In contrast to the criterion #7 above which assesses barriers and facilitators as inputs to developing the intervention strategy, this criterion assesses the actual barriers and facilitators identified during and after the implementation.  (From CReDECI, Mohler, 2012; also mentioned in Michie, 2009)  **Explanation/Example:**  "The attitudes of the nursing home managers turned out to be an important factor supporting or impeding the success of the intervention's implementation. The more the managers agreed with the interventions’ aim, the better the nursing staff felt supported." | Physical space was a critical constraint in the expansion of this programme, as the clinic had to be relocated twice in 3 years, including the development of an off-site location for pre-treatment counselling. In addition, the positioning of a dedicated laboratory in a shipping container on site enabled an efficient monitoring service, avoiding the need for sample courier services and expediting access of results to medical  staff. | Good |
| **Criterion #9**  **Description of materials:** Description of all materials or tools used for the implementation  (From CReDECI, Mohler, 2012)  **Explanation/Example:**  "The primary enablers of behaviour change were paid community-based health workers, who were recruited from the local community based on 12 years or more of education,  proficient communication and reasoning skills, commitment towards community work, and references of community stakeholders. They received a combination of class room based  and apprentice ship-based field training over 7 days on knowledge, attitudes, and practices related to essential newborn care within the community, behaviour change management, and trust-building. After training, suitable candidates were closely mentored and supervised by a regional programme supervisor (n=4) responsible for 6–7 trainees, for an additional week before final selection was made." | Medications, facilities, and background of counselors was well described. However, no “materials” such as educational pamphlets were described. | Fair |
| **Criterion #10**  **Process of Implementation:** Description of an assessment of the implementation process  (From CReDECI, Mohler 2012)  **Explanation/Example:**  Process assessment is a prerequisite for determining the success of the intervention's implementation and should be an integral part of an assessment of the intervention’s effect. For example, "To gain insight into the dissemination and the delivery of the  intervention and to draw conclusions about potential barriers and facilitators to implementing the intervention in other settings, data on the implementation process were collected alongside the randomized-controlled trial. Therefore, we assessed the quality of  delivery of the interventional components (observed by members of the research team not involved in the delivery of the intervention) and the adherence to study protocol (number  and type of deviations from the protocol, using a pilot-tested standardized form). We also analyzed barriers and facilitators for the delivery of intervention’s components (focus  group interviews with intervention participants)." | At the screening visit, a treatment-readiness evaluation was completed and a 4-week supply of cotrimoxazole was dispensed to patients with less than 200 CD4 cells/μl, with pill counts at 14 and 28 days to assess  adherence. Plasma HIV load and blood CD4 cell count were performed  at week 2 and 4-monthly after commencing ART. Structured clinical records were maintained on all patients … and this information  together with laboratory results was regularly transferred to an electronic database... for programme evaluation | Poor |

**Lay Health Workers**

**Additional File 2-I. Criterion Table for Sloan et al.; 2008**

| **Criterion** | **Example of text related to this criterion** | **Rating** |
| --- | --- | --- |
| **Criterion #1**  **Intervention Characteristics:** Intervention/Program source (From CFIR, Damschroder, 2009)^2^  **Explanation/Example:**  Is the intervention/program externally or internally developed? An intervention/program may be internally developed as a good idea, a solution to a problem, or other grass roots effort, or may be developed by an external entity (such as a foundation or a NGO). Interventions or programs that arise internally from the populations who will be impacted are sometimes more sustainable than externally developed programs dependent on external funding. The perceived legitimacy of the source may also influence implementation. | Together with the Bangladesh Rural Advancement Committee (BRAC), Mitra and Associates, Ecuadorian and Bangladeshi physicians, nurse-midwives, and KMC experts, the study team adapted KMC so that it can be feasibly implemented as a community-based intervention (CKMC).  (*This seems to imply that the intervention was externally developed)* | Good |
| **Criterion #2**  **Intervention Characteristics:** A description of why the intervention was hypothesized to have an impact on the outcome, according to theory. (From CReDECI, Mohler 2012; also mentioned in Michie, 2009)^3,4^  **Explanation/Example:**  The theoretical basis of the intervention should be clearly stated. This includes the theory on which the intervention is founded as well as, if available, empirical evidence from studies in different settings or countries. For example, "The implementation was based on Rogers’ Diffusion of Innovation theory, which posits 5 factors of innovation that influence a decision to adopt or reject an innovation: relative advantage, compatibility, complexity or simplicity, trialability, observability. A similar intervention, also based on Rogers’ Diffusion of Innovation theory, was successfully implemented in other countries." | Kangaroo mother care (KMC) is a method whereby the hospital-born stabilized LBW newborn is placed in skin-to-skin (STS) contact on the mother’s breast to promote thermal regulation, breastfeeding, and maternal–infant bonding. Traditional KMC reduces the incidence of morbidity but not mortality in LBW infants, because it is generally applied to clinically stabilized newborns and most neonatal mortality occurs in the first 2 days of life before stabilization. A single adequately designed study found a 43% (not statistically significant) lower infant mortality rate (IMR) associated with traditional KMC. Two small African studies of early (as soon as possible after birth) KMC in hospitals with little neonatal intensive care capacity reported reduced mortality within 24 hours of birth and before discharge; however, important differences in study group characteristics were not controlled in analysis.  *(Describes the rational for why the intervention should work (Thermoregulation, breast feeding, bonding) and describes prior data from similar studies)* | Poor / None |
| **Criterion #3**  **Intervention Characteristics:**  Rationale for the aim/essential functions of the intervention/program’s components, including the evidence whether the components are appropriate for achieving this goal.  This differs from the need to articulate the theory behind the intervention in that the theory posits the general principles (such as Rogers Diffusion of Innovation) while this item is about specific components of the intervention and the effects of the component on specific targets. (From CReDECI, Mohler, 2012; also mentioned in Michie, 2009)^3,4^ | No text was found. | Poor / None |
| **Criterion #4**  **Intervention Characteristics:**  Detailed description of the intervention/program (From WIDER as described in Michie, 2009)^4^  **The detailed description should include:**  a. Characteristics of those delivering the intervention/program (such as a nurse or lay health worker)  b. Characteristics of the recipients  c. The setting  d. The mode of delivery (such as face-to-face)  e. The intensity of the intervention/program (such as the contact time with participants)  f. The duration (such as the number of sessions and their spacing interval over a given period) | a. A physician who had participated in the pilot study trained 12 BRAC  supervisors and, along with 1 supervisor, trained all 63 community nutrition workers and their 25 NNP supervisors serving the intervention group in 5 groups of 6 to 22 people during a 2-month period.  b. A detailed table of characteristics of the recipients is included in Table 1.  c. Bangladesh has a population of 140 000 000 administratively divided into  6 divisions that, combined, contain 64 districts and 496 subdistricts called upazilas, each of which has a capitol city. Each study subdistrict contains 8 to 14 unions, and each union contains 5 to 25 villages. The sample includes the 42 unions that participated in the NNP, all that are supervised by our study partner BRAC in the Dhaka and Sylhet divisions. Dhaka and Sylhet divisions are located in northern Bangladesh, where NMR was 5.2% and 8.2% and IMR was 11.5% and 16.2%, the highest in the nation, when the study was designed.  d. Although not stated, it is implied that the intervention is developed face-to-face.  e. No text was found.  f. No text was found. | Fair  Good  Good  Fair  Poor / None  Poor / None |
| g. Adherence or fidelity to delivery protocols | g. The training and intervention delivery processes that were used in the pilot study were only partially transferred to the full trial, with unplanned substitution of experienced trainers with individuals who were not trainers, less frequent contact between community workers and mothers in the last month of pregnancy, and unplanned emphasis on CKMC for LBW infants.  The nested qualitative study found that >35% of CKMC women were erroneously taught that STS was to be provided to LBW or preterm infants rather than to all infants, and only 30% were correctly taught to hold all  infants STS. Less than 40% of CKMC mothers were taught to provide CKMC to infants who were ill, and >25% were erroneously taught to breastfeed on schedule (not on demand). Women had numerous views  about the number of hours and days they should provide STS, indicating that they received variable and frequently incorrect messages from the community workers and supervisors. | Good |
| **Criterion #5**  **Intervention Characteristics:**  Costs of the intervention and costs associated with implementing the intervention (From CFIR, Damschroder, 2009; CReDECI, Mohler, 2012)^2,3^  **Explanation/Example:**  The cost of the intervention and implementation can influence the adoption and sustainability; interventions maybe more difficult to sustain if they were supported as part of a research study. | All participating community workers received $7.50 a month.  *(Only cost data identified)* | Poor / None |
| **Criterion #6**  **Outer Setting:** External policies and incentives (From CFIR, Damschroder, 2009)^2^  **Explanation/Example:**  How does the health service, intervention, or program relate to country and global health goals? Is the program part of a larger strategy? If so how is it strategically aligned? A country's health policies may influence the implementation of a particular intervention or program. | Not explicitly stated. | Poor / None |
| **Criterion #7**  **Population needs**  (From CFIR, Damschroder, 2009)^2^  **Explanation/Example:**  The extent to which population needs, as well as barriers and facilitators to meet those needs, are accurately known and prioritized. This could include population-based data on causes of morbidity and mortality, political or cultural barriers or facilitators, and/or more locally focused data about local needs, barriers or facilitators. | No text was found. | Poor / None |
| **Criterion #8**  **Process of implementation:** Description of facilitators or barriers which have influenced the intervention or program’s implementation (see #10) revealed by a process assessment.  In contrast to the criterion #7 above which assesses barriers and facilitators as inputs to developing the intervention strategy, this criterion assesses the actual barriers and facilitators identified during and after the implementation.  (From CReDECI, Mohler, 2012; also mentioned in Michie, 2009)^3,4^  **Explanation/Example:**  "The attitudes of the nursing home managers turned out to be an important factor supporting or impeding the success of the intervention's implementation. The more the managers agreed with the interventions’ aim, the better the nursing staff felt supported." | Field visits confirmed that some of those who were employed to conduct the CKMC training believed that CKMC was intended for small infants.  Thus, some intervention group mothers may not have provided CKMC because they were mistakenly taught that CKMC is for small infants. CKMC implementation and effect depend on both the quality of CKMC training and the mother’s behavior modification, making it difficult to know whether the intervention does not have effect in larger, more mature infants or whether the uptake was suboptimal as a result of insufficient training or poor maternal adherence.  *(This indicates that insufficiently trained CKMC trainers decreased the effectiveness of the intervention)* | Good |
| **Criterion #9**  **Description of materials:** Description of all materials or tools used for the implementation  (From CReDECI, Mohler, 2012)^3^  **Explanation/Example:**  "The primary enablers of behaviour change were paid community-based health workers, who were recruited from the local community based on 12 years or more of education,  proficient communication and reasoning skills, commitment towards community work, and references of community stakeholders. They received a combination of classroombased and apprentice ship-based field training over 7 days on knowledge, attitudes, and practices related to essential newborn care within the community, behaviour change management, and trust-building. After training, suitable candidates were closely mentored and supervised by a regional programme supervisor (n=4) responsible for 6–7 trainees, for an additional week before final selection was made." | No text was found. | Poor / None |
| **Criterion #10**  **Process of Implementation:** Description of an assessment of the implementation process  (From CReDECI, Mohler 2012)^3^  **Explanation/Example:**  Process assessment is a prerequisite for determining the success of the intervention's implementation and should be an integral part of an assessment of the intervention’s effect. For example, "To gain insight into the dissemination and the delivery of the intervention and to draw conclusions about potential barriers and facilitators to implementing the intervention in other settings, data on the implementation process were collected alongside the randomized-controlled trial. Therefore, we assessed the quality of delivery of the interventional components (observed by members of the research team not involved in the delivery of the intervention) and the adherence to study protocol (number and type of deviations from the protocol, using a pilot-tested standardized form). We also analyzed barriers and facilitators for the delivery of intervention’s components (focus group interviews with intervention participants)." | The training and intervention delivery processes that were used in the pilot study were only partially transferred to the full trial, with unplanned substitution of experienced trainers with individuals who were not trainers, less frequent contact between community workers and mothers in the last month of pregnancy, and unplanned emphasis on CKMC for LBW infants.  The nested qualitative study found that >35% of CKMC women were erroneously taught that STS was to be provided to LBW or preterm infants rather than to all infants, and only 30% were correctly taught to hold all  infants STS. Less than 40% of CKMC mothers were taught to provide CKMC to infants who were ill, and >25% were erroneously taught to breastfeed on schedule (not on demand). Women had numerous views  about the number of hours and days they should provide STS, indicating that they received variable and frequently incorrect messages from the community workers and supervisors.  *(Indicates implementation was not as successful as planned)* | Good |

**Additional File 2-J. Criterion Table for Kouyate et al.; 2008**

| **Criterion** | **Example of text related to this criterion** | **Rating** |
| --- | --- | --- |
| **Criterion #1**  **Intervention Characteristics:** Intervention/Program source (From CFIR, Damschroder, 2009)^2^  **Explanation/Example:**  Is the intervention/program externally or internally developed? An intervention/program may be internally developed as a good idea, a solution to a problem, or other grass roots effort, or may be developed by an external entity (such as a foundation or a NGO). Interventions or programs that arise internally from the populations who will be impacted are sometimes more sustainable than externally developed programs dependent on external funding. The perceived legitimacy of the source may also influence implementation. | This project is an EU INCO-DEV funded collaboration between the Heidelberg University (Germany), Karolinska Institute (Sweden), Muhimbili University College of Health Sciences (Tanzania) and Centre de Recherche en Santé de Nouna (Burkina Faso) called MAMOP project  (Improving the management of childhood Malaria: an experiment to bridge the gap between Mothers and health care Providers).  *(Indicates the intervention was developed externally)* | Good |
| **Criterion #2**  **Intervention Characteristics:** A description of why the intervention was hypothesized to have an impact on the outcome, according to theory. (From CReDECI, Mohler 2012; also mentioned in Michie, 2009)^3,4^  **Explanation/Example:**  The theoretical basis of the intervention should be clearly stated. This includes the theory on which the intervention is founded as well as, if available, empirical evidence from studies in different settings or countries. For example, "The implementation was based on Rogers’ Diffusion of Innovation theory, which posits 5 factors of innovation that influence a decision to adopt or reject an innovation: relative advantage, compatibility, complexity or simplicity, trialability, observability. A similar intervention, also based on Rogers’ Diffusion of Innovation theory, was successfully implemented in other countries." | No text was found. | Poor / None |
| **Criterion #3**  **Intervention Characteristics:**  Rationale for the aim/essential functions of the intervention/program’s components, including the evidence whether the components are appropriate for achieving this goal.  This differs from the need to articulate the theory behind the intervention in that the theory posits the general principles (such as Rogers Diffusion of Innovation) while this item is about specific components of the intervention and the effects of the component on specific targets. (From CReDECI, Mohler, 2012; also mentioned in Michie, 2009)^3,4^ | No text was found. | Poor / None |
| **Criterion #4**  **Intervention Characteristics:**  Detailed description of the intervention/program (From WIDER as described in Michie, 2009)^4^  **The detailed description should include:**  a. Characteristics of those delivering the intervention/program (such as a nurse or lay health worker)  b. Characteristics of the recipients  c. The setting | a. Inclusion criteria for group leaders used by the communities were permanent residency in the sub-village, age 30–50 years, honesty, and respect by the community. A five days training course for the health workers of participating health centres was conducted by one of the investigators (FS).  b. The intervention was targeted at three groups: health workers (nurses) from five peripheral health centres (Toni, Dara, Bourasso, Lekuy, Koro), women group leaders, and the main care takers (usually the mothers) of preschool children.  c. The study was implemented in the rural part of the research zone of the Centre de Recherche en Santé de Nouna (CRSN) in Nouna Health District, north-western Burkina Faso (Figure 1). The Nouna area is a dry orchard savannah, populated mainly by subsistence farmers of different ethnic groups. Malaria is holoendemic but highly seasonal, and the transmission intensity varies between 100 and 1000 infective bites per person and year between study villages. Formal health services in the study area are provided by a limited number of rural health centres and the district hospital in Nouna town. Villagebased health centres are usually equipped with two nurses and one mid-wife and do outreach work in the surrounding surrounding 7–10 villages under their responsibility. Malaria control is mainly based on home treatment with CQ, which has been shown to be still sufficiently effective in 2001, and on malaria prophylaxis for pregnant women. Untreated mosquito nets have been used in the area for a long time, but insecticide-treated nets (ITN) were only recently introduced in the frame of an effectiveness study. Communities in the study area have been shown to be quite well organized with regard to risk sharing mechanisms. In particular women groups with a focus on mutual agricultural support traditionally exist in all villages. | Fair  Fair  Good |
| d. The mode of delivery (such as face-to-face)  e. The intensity of the intervention/program (such as the contact time with participants)  f. The duration (such as the number of sessions and their spacing interval over a given period)  g. Adherence or fidelity to delivery protocols | d. Although not stated, it is implied that the intervention is developed face-to-face.  e. No text was found.  f. No text was found.  g. The only data on adherence or fidelity to delivery protocols was self-reported treatment with chloroquine listed in Table 4.  Some components are described in great detail. Reproductions of charts and pictures provided to recipients are presented, however other components such as the training given to women group leaders were only briefly described or not at all. | Fair  Poor / None  Poor / None  Fair |
| **Criterion #5**  **Intervention Characteristics:**  Costs of the intervention and costs associated with implementing the intervention (From CFIR, Damschroder, 2009; CReDECI, Mohler, 2012)^2,3^  **Explanation/Example:**  The cost of the intervention and implementation can influence the adoption and sustainability; interventions maybe more difficult to sustain if they were supported as part of a research study. | The only cost data reported was the cost of chloroquine. | Poor / None |
| **Criterion #6**  **Outer Setting:** External policies and incentives (From CFIR, Damschroder, 2009)^2^  **Explanation/Example:**  How does the health service, intervention, or program relate to country and global health goals? Is the program part of a larger strategy? If so how is it strategically aligned? A country's health policies may influence the implementation of a particular intervention or program. | Not explicitly stated. | Poor / None |
| **Criterion #7**  **Population needs**  (From CFIR, Damschroder, 2009)^2^  **Explanation/Example:**  The extent to which population needs, as well as barriers and facilitators to meet those needs, are accurately known and prioritized. This could include population-based data on causes of morbidity and mortality, political or cultural barriers or facilitators, and/or more locally focused data about local needs, barriers or facilitators. | The only description of population needs was that malaria is holoendemic to the area. | Poor / None |
| **Criterion #8**  **Process of implementation:** Description of facilitators or barriers which have influenced the intervention or program’s implementation (see #10) revealed by a process assessment.  In contrast to the criterion #7 above which assesses barriers and facilitators as inputs to developing the intervention strategy, this criterion assesses the actual barriers and facilitators identified during and after the implementation.  (From CReDECI, Mohler, 2012; also mentioned in Michie, 2009)^3,4^  **Explanation/Example:**  "The attitudes of the nursing home managers turned out to be an important factor supporting or impeding the success of the intervention's implementation. The more the managers agreed with the interventions’ aim, the better the nursing staff felt supported." | No text found. | Poor / None |
| **Criterion #9**  **Description of materials:** Description of all materials or tools used for the implementation  (From CReDECI, Mohler, 2012)^3^  **Explanation/Example:**  "The primary enablers of behaviour change were paid community-based health workers, who were recruited from the local community based on 12 years or more of education,  proficient communication and reasoning skills, commitment towards community work, and references of community stakeholders. They received a combination of classroombased and apprentice ship-based field training over 7 days on knowledge, attitudes, and practices related to essential newborn care within the community, behaviour change management, and trust-building. After training, suitable candidates were closely mentored and supervised by a regional programme supervisor (n=4) responsible for 6–7 trainees, for an additional week before final selection was made." | Some components are described in great detail. Reproductions of charts and pictures provided to recipients are presented, however other components such as the training given to women group leaders were only briefly described or not at all. | Fair |
| **Criterion #10**  **Process of Implementation:** Description of an assessment of the implementation process  (From CReDECI, Mohler 2012)^3^  **Explanation/Example:**  Process assessment is a prerequisite for determining the success of the intervention's implementation and should be an integral part of an assessment of the intervention’s effect. For example, "To gain insight into the dissemination and the delivery of the intervention and to draw conclusions about potential barriers and facilitators to implementing the intervention in other settings, data on the implementation process were collected alongside the randomized-controlled trial. Therefore, we assessed the quality of delivery of the interventional components (observed by members of the research team not involved in the delivery of the intervention) and the adherence to study protocol (number and type of deviations from the protocol, using a pilot-tested standardized form). We also analyzed barriers and facilitators for the delivery of intervention’s components (focus group interviews with intervention participants)." | No text found. | Poor / None |

**Additional File 2-K. Criterion Table for Kumar et al.; 2008**

| **Criterion** | **Example of text related to this criterion** | **Rating** |
| --- | --- | --- |
| **Criterion #1**  **Intervention Characteristics:** Intervention/Program source (From CFIR, Damschroder, 2009)^2^  **Explanation/Example:**  Is the intervention/program externally or internally developed? An intervention/program may be internally developed as a good idea, a solution to a problem, or other grass roots effort, or may be developed by an external entity (such as a foundation or a NGO). Interventions or programs that arise internally from the populations who will be impacted are sometimes more sustainable than externally developed programs dependent on external funding. The perceived legitimacy of the source may also influence implementation. | We postulated that an intervention based on a socioculturally contextualised approach of behavior change management systematically applied to modifiable, high-risk newborn-care practices, with an emphasis on hypothermia, within a community with a high neonatal mortality rate could lead to improved care practices and reduced mortality.  (*This seems to indicate the intervention was developed by the research teams – but later, there is this text:*  Qualitative research activities provided the evidence base for investigators and community members to codevelop the intervention strategy, which underwent further refinement based on findings of trials of improved practices.  *so should this be considered internally developed?)* | Fair |
| **Criterion #2**  **Intervention Characteristics:** A description of why the intervention was hypothesized to have an impact on the outcome, according to theory. (From CReDECI, Mohler 2012; also mentioned in Michie, 2009)^3,4^  **Explanation/Example:**  The theoretical basis of the intervention should be clearly stated. This includes the theory on which the intervention is founded as well as, if available, empirical evidence from studies in different settings or countries. For example, "The implementation was based on Rogers’ Diffusion of Innovation theory, which posits 5 factors of innovation that influence a decision to adopt or reject an innovation: relative advantage, compatibility, complexity or simplicity, trialability, observability. A similar intervention, also based on Rogers’ Diffusion of Innovation theory, was successfully implemented in other countries." | In a study in Maharashtra, India, Bang and colleagues reported a 62–70% reduction in the neonatal mortality rate, and attributed 93% of the reduction to active management of sick newborn babies and 7% to primary prevention. Baqui and colleagues reported that an adaptation of this approach in Bangladesh in an effectiveness trial had half the effect (34% reduction) on neonatal mortality. Manandhar and co-workers3 tested a different approach in Nepal with a community-based participatory action-cycle with no prespecified intervention package, in which women’s groups identified priorities and implemented local solutions, and reported improvements in care practices, care-seeking, and a 30% reduction in neonatal mortality rate.  We postulated that an intervention based on a socioculturally contextualised approach of behavior change management systematically applied to modifiable, high-risk newborn-care practices, with an emphasis on hypothermia, within a community with a high neonatal mortality rate could lead to improved care practices and reduced mortality.  *(Identifies both a framework for why it should work and prior data)* | Good |
| **Criterion #3**  **Intervention Characteristics:**  Rationale for the aim/essential functions of the intervention/program’s components, including the evidence whether the components are appropriate for achieving this goal.  This differs from the need to articulate the theory behind the intervention in that the theory posits the general principles (such as Rogers Diffusion of Innovation) while this item is about specific components of the intervention and the effects of the component on specific targets. (From CReDECI, Mohler, 2012; also mentioned in Michie, 2009)^3,4^ | Our preliminary qualitative field work showed that individual behaviours were influenced by collective behaviours and social norms, and sustained by a complex, multilevel network of relationships within the community. We therefore developed a multilevel strategy targeting: community stakeholders, newborn stake holders, and households with immediate support groups. At each level, the target group consisted of individuals who were identified to have key roles as influencers, decision makers, supporters, and practitioners of newborn care and normative behaviour within the community. The support of community stake holders such as village heads, community leaders, respected members, priests, and teachers was crucial in building trust with the community and ensuring acceptance of the programme. The newborn stakeholder target group included traditional newborn-care providers and birth attendants, unqualified medical practitioners, and, to a lesser extent, health system workers, some of whom had strategic access to the newborn and mother during post-partum confinement, were perceived by the community as domain experts, and played an active part in sustaining targeted practices. Health system workers such as auxillary nurse midwives were engaged only at the community level as part of newborn stakeholder group meetings in order to keep contamination of the intervention into control clusters to a minimum. The household target group included the pregnant woman or mother, who was the primary care provider, but usually not empowered to make decisions; the mother-in-law, who was usually the key decision maker on newborn-care practices; other female members who played supportive roles; and male members, including the father-in-law and husband, who controlled access to the household, made financial and logistical arrangements, and influenced care-seeking decisions. The family’s immediate support group included neighbours and relatives who influenced family behaviours and helped with deliveries.  *(Indicates extensive field work to fit intervention components to the needs of the population)* | Good |
| **Criterion #4**  **Intervention Characteristics:**  Detailed description of the intervention/program (From WIDER as described in Michie, 2009)^4^  **The detailed description should include:**  a. Characteristics of those delivering the intervention/program (such as a nurse or lay health worker)  b. Characteristics of the recipients  c. The setting  d. The mode of delivery (such as face-to-face)  e. The intensity of the intervention/program (such as the contact time with participants)  f. The duration (such as the number of sessions and their spacing interval over a given period)  g. Adherence or fidelity to delivery protocols | a. The primary enablers of behaviour change were paid (US$35–40 per month) community-based health workers, the Saksham Sahayak (n=26), who were recruited from the local community based on 12 years or more of education, proficient communication and reasoning skills, commitment towards community work, and references of community stakeholders.  b. At each level, the target group consisted of individuals who were identified to have key roles as influencers, decision makers, supporters, and practitioners of newborn care and normative behaviour within the community.  c. The state of Uttar Pradesh, India, accounts for a quarter of India’s neonatal deaths and for 8% of those worldwide, and shares similar sociocultural, demographic, and health system characteristics with other high-mortality Indian states and south Asian countries. The study was done in Shivgarh, a rural block in Uttar Pradesh, with a population of 104 123 divided into 39 village administrative units . Socioeconomic indicators are among the lowest in the state. The formal health-care system in Shivgarh consists of a community health centre and two primary health centres operated by trained physicians and paramedical staff supported by 18 auxiliary nurse midwives, who are outreach workers catering to a population of 6000–7000 each, and trained to deliver babies, and provide vaccinations and antenatal check-ups. Care-seeking from them, however, is low.  d. The intervention was delivered from January, 2004, to May, 2005. Saksham Sahayaks first engaged with community stakeholders in community meetings to seek their approval, sensitise them towards the importance of their role in newborn survival, encourage shared learning, and create a supportive environment (figure 1, webtable 2). Folk song group meetings, where messages to promote behaviour change were incorporated into folk songs, were held by Saksham Sahayaks on a monthly basis with participants from diverse target groups. They also held separate monthly meetings with newborn-care stakeholders and with community volunteers to discuss experiences, challenges, and strategies.  e. This process was accomplished through 3-monthly cycles of door-to-door household visits by Saksham Sahayaks, self-reporting by pregnant women, and information provided by community volunteers. An antenatal visit was planned for 60 days before the expected date of delivery and another for 30 days before the expected date of delivery to provide ample time for effective behaviour change negotiation, ensure birth preparedness, and build trust with the family to negotiate subsequent entry into the room of confinement after delivery for postnatal visits (webtable 3). Post-partum confinement was a universal practice, and coincided with the initiation of almost all the targeted practices and occurrence of most newborn deaths. As some of the new practices were skill-based,  f. The first postnatal visit was planned within 24 h of the delivery and the second postnatal visit was planned on day 3 (webtable 3). In case of sick neonates, no treatment was provided, but families were advised to seek care at the nearest health facility.  g. Coverage of household visits by *Saksham Sahayaks* was calculated as the ratio of total visitations recorded during the study period to the total number of women eligible for the visitations. For coverage on antenatal visits, all pregnancies were considered eligible and for coverage on postnatal visits, all women with at least one liveborn baby were considered eligible for the visits. Household visits by newborn-care stakeholders and community volunteers in the absence of *Saksham Sahayaks* were not recorded. The monthly coverage of group meetings was based on monitoring reports by *Saksham Sahayaks*. | Good  Good  Good  Good  Good  Good  Good |
| **Criterion #5**  **Intervention Characteristics:**  Costs of the intervention and costs associated with implementing the intervention (From CFIR, Damschroder, 2009; CReDECI, Mohler, 2012)^2,3^  **Explanation/Example:**  The cost of the intervention and implementation can influence the adoption and sustainability; interventions maybe more difficult to sustain if they were supported as part of a research study. | The primary enablers of behaviour change were paid (US$35–40 per month) community-based health workers.  *(Only cost data identified)* | Poor / None |
| **Criterion #6**  **Outer Setting:** External policies and incentives (From CFIR, Damschroder, 2009)^2^  **Explanation/Example:**  How does the health service, intervention, or program relate to country and global health goals? Is the program part of a larger strategy? If so how is it strategically aligned? A country's health policies may influence the implementation of a particular intervention or program. | Not explicitly mentioned | None |
| **Criterion #7**  **Population needs**  (From CFIR, Damschroder, 2009)^2^  **Explanation/Example:**  The extent to which population needs, as well as barriers and facilitators to meet those needs, are accurately known and prioritized. This could include population-based data on causes of morbidity and mortality, political or cultural barriers or facilitators, and/or more locally focused data about local needs, barriers or facilitators. | Participatory social mapping of all villages in the study area provided an  introduction to the community, initiated the process of collaborative engagement, served to identify community resources for newborn health, and facilitated the planning of home visitations and group interventions.  Qualitative research activities provided the evidence base for investigators and community members to codevelop the intervention strategy, which underwent further refinement based on findings of trials of improved practices.  Domiciliary care practices were mapped against the existing evidence base of risk factors for neonatal mortality and morbidity. Practices that were assessed to be potentially harmful, preventable, within community control, and amenable to change were selected for behavioural modification (webtable 1).  *(Field work to identify potential barriers and facilitators of implantation)* | Good |
| **Criterion #8**  **Process of implementation:** Description of facilitators or barriers which have influenced the intervention or program’s implementation (see #10) revealed by a process assessment.  In contrast to the criterion #7 above which assesses barriers and facilitators as inputs to developing the intervention strategy, this criterion assesses the actual barriers and facilitators identified during and after the implementation.  (From CReDECI, Mohler, 2012; also mentioned in Michie, 2009)^3,4^  **Explanation/Example:**  "The attitudes of the nursing home managers turned out to be an important factor supporting or impeding the success of the intervention's implementation. The more the managers agreed with the interventions’ aim, the better the nursing staff felt supported." | No text was found about facilitators and barriers to the actual implementation. | Poor / None |
| **Criterion #9**  **Description of materials:** Description of all materials or tools used for the implementation  (From CReDECI, Mohler, 2012)^3^  **Explanation/Example:**  "The primary enablers of behaviour change were paid community-based health workers, who were recruited from the local community based on 12 years or more of education,  proficient communication and reasoning skills, commitment towards community work, and references of community stakeholders. They received a combination of classroombased and apprentice ship-based field training over 7 days on knowledge, attitudes, and practices related to essential newborn care within the community, behaviour change management, and trust-building. After training, suitable candidates were closely mentored and supervised by a regional programme supervisor (n=4) responsible for 6–7 trainees, for an additional week before final selection was made." | Available in a web appendix. | Good |
| **Criterion #10**  **Process of Implementation:** Description of an assessment of the implementation process  (From CReDECI, Mohler 2012)^3^  **Explanation/Example:**  Process assessment is a prerequisite for determining the success of the intervention's implementation and should be an integral part of an assessment of the intervention’s effect. For example, "To gain insight into the dissemination and the delivery of the intervention and to draw conclusions about potential barriers and facilitators to implementing the intervention in other settings, data on the implementation process were collected alongside the randomized-controlled trial. Therefore, we assessed the quality of delivery of the interventional components (observed by members of the research team not involved in the delivery of the intervention) and the adherence to study protocol (number and type of deviations from the protocol, using a pilot-tested standardized form). We also analyzed barriers and facilitators for the delivery of intervention’s components (focus group interviews with intervention participants)." | No text was found describing the assessment of implementation. | Poor / None |

**Additional File 2-L. Criterion Table for Bari et al.; 2006**

| **Criterion** | **Example of text related to this criterion** | **Rating** |
| --- | --- | --- |
| **Criterion #1**  **Intervention Characteristics:** Intervention/Program source  (From CFIR, Damschroder, 2009)  **Explanation/Example:**  Is the intervention/program externally or internally developed? An intervention/program may be internally developed as a good idea, a solution to a problem, or other grass roots effort, or may be developed by an external entity (such as a foundation or a NGO). Interventions or programs that arise internally from the populations who will be impacted are sometimes more sustainable than externally developed programs dependent on external funding. The perceived legitimacy of the source may also influence implementation. | This study was supported primarily through the generous support of the Infectious Disease Initiative of the Wellcome Trust–Burroughs Wellcome Fund. Additional support was provided by the Department for International Development (DFID), UK; the United States Agency for International Development, Office of Health, Infectious Diseases, and Nutrition, Global Bureau through the Global Research Activity Cooperative Agreement (No. GHS-A-00-03-00019-00); the Government of Bangladesh (Improved Health for the Poor); and Save the Children-USA through a grant from the Bill and Melinda Gates Foundation. (Indicates this is externally developed) | Good |
| **Criterion #2**  **Intervention Characteristics:** A description of why the intervention was hypothesized to have an impact on the outcome, according to theory. (From CReDECI, Mohler 2012; also mentioned in Michie, 2009)  **Explanation/Example:**  The theoretical basis of the intervention should be clearly stated. This includes the theory on which the intervention is founded as well as, if available, empirical evidence from studies in different settings or countries. For example, "The implementation was based on Rogers’ Diffusion of Innovation theory, which posits 5 factors of innovation that influence a decision to adopt or reject an innovation: relative advantage, compatibility, complexity or simplicity, trialability, observability. A similar intervention, also based on Rogers’ Diffusion of Innovation theory, was successfully implemented in other countries." | First, there is a need to act rapidly for certain maternal and newborn conditions, such as severe postpartum haemorrhage and birth asphyxia. So, research needs to examine not only recognition of danger signs, but also how long it takes for recognition of the problem to occur.  Second, in both mothers and newborns, many important causes of morbidity and mortality have signs and symptoms that “lie on a continuum, from normal to abnormal” (16). For such signs and symptoms, there can be both over-reporting of non-serious conditions, such as transient tachypnoea of the newborn or upper respiratory viral infection, or under-reporting of truly  serious conditions, as happens when families fail to recognize excessive loss of maternal blood after delivery (16). Finally, patterns of decision-making within the household may lead to a significant delay, especially when permission of the husband is needed before seeking care but he is away, or when it is not clear to the family where to seek care from among a range of formal and informal sector providers (17-19). | Good |
| **Criterion #3**  **Intervention Characteristics:**  Rationale for the aim/essential functions of the intervention/program’s components, including the evidence whether the components are appropriate for achieving this goal.  This differs from the need to articulate the theory behind the intervention in that the theory posits the general principles (such as Rogers Diffusion of Innovation) while this item is about specific components of the intervention and the effects of the component on specific targets.  (From CReDECI, Mohler, 2012; also mentioned in Michie, 2009) | During the antenatal period, families were educated about signs indicating the need to seek care and sources of care by health workers during antenatal care visits at health facilities and by CHWs during antenatal home-visits (left side of Fig. 1). This should lead to an increased recognition by families of danger signs in newborns. This, in turn, should lead them either to seek  care directly (self-referral) from the Kumudini Hospital and other appropriate sources of care, or notify the CHW to come to the house and assess the child. | Fair |
| **Criterion #4**  **Intervention Characteristics:**  Detailed description of the intervention/program (From WIDER as described in Michie, 2009)  **The detailed description should include:**  a. Characteristics of those delivering the intervention/program (such as a nurse or lay health worker)  b. Characteristics of the recipients  c. The setting  d. The mode of delivery (such as face-to-face)  e. The intensity of the intervention/program (such as the contact time with participants)  f. The duration (such as the number of sessions and their spacing interval over a given period)  g. Adherence or fidelity to delivery protocols  h. A detailed description of the intervention/program content provided to each study group | a. These CHWs had a minimum of 10th grade education and resided in the population they would serve.  b. No text found  c. The Projahnmo-II Project in Mirzapur upazila (subdistrict) of Tangail district in central Bangladesh Mirzapur upazila has 13 unions, with a population of around 24,000 each;  d. and made home-visits in the third and the eighth month of pregnancy  e. and made home-visits in the third and the eighth month of pregnancy, on day 0, 3, 6, and 9.  f. (day of birth), 3, 6, and 9.  g. Of 3,354 women who did not change residence, 3,228 (96.2%) received at least one home-visit by the CHW during the first 28 days of life of the baby.  h. No text found | Good  Poor  Fair  Fair  Good  Good  Fair  Poor |
| **Criterion #5**  **Intervention Characteristics:**  Costs of the intervention and costs associated with implementing the intervention (From CFIR, Damschroder, 2009; CReDECI, Mohler, 2012)  **Explanation/Example:**  The cost of the intervention and implementation can influence the adoption and sustainability; interventions maybe more difficult to sustain if they were supported as part of a research study. | No text found | Poor |
| **Criterion #6**  **Outer Setting:** External policies and incentives (From CFIR, Damschroder, 2009)  **Explanation/Example:**  How does the health service, intervention, or program relate to country and global health goals? Is the program part of a larger strategy? If so how is it strategically aligned? A country's health policies may influence the implementation of a particular intervention or program. | No text found | Poor |
| **Criterion #7**  **Population needs**  (From CFIR, Damschroder, 2009)  **Explanation/Example:**  The extent to which population needs, as well as barriers and facilitators to meet those needs, are accurately known and prioritized. This could include population-based data on causes of morbidity and mortality, political or cultural barriers or facilitators, and/or more locally focused data about local needs, barriers or facilitators. | In many cultures, families practise a period of postpartum confinement  of both mother and newborn lasting from one to six week(s) or more (20). Additional efforts may be needed during this period to convince families to seek care outside the home. | Fair |
| **Criterion #8**  **Process of implementation:** Description of facilitators or barriers which have influenced the intervention or program’s implementation (see #10) revealed by a process assessment.  In contrast to the criterion #7 above which assesses barriers and facilitators as inputs to developing the intervention strategy, this criterion assesses the actual barriers and facilitators identified during and after the implementation.  (From CReDECI, Mohler, 2012; also mentioned in Michie, 2009)  **Explanation/Example:**  "The attitudes of the nursing home managers turned out to be an important factor supporting or impeding the success of the intervention's implementation. The more the managers agreed with the interventions’ aim, the better the nursing staff felt supported." | The reasons cited for non-compliance (multiple responses allowed) included: nobody was available to accompany the child (and the mother) to the health facility (24.7%); the child was given a traditional treatment instead (19.1%); bad weather or general strikes (17.9%); the family disliked hospital treatment (12.3%); symptoms resolved on their own (7.4%); unwillingness of the family or the TBA to refer the baby for other reasons (6.2%); and other issues (12.3%), such as illness of the mother; the child was too young to be taken for outside care; and lack of transport.  Substantial increases in referral compliance for newborn illness were likely related to (a) education of families on danger signs by the CHWs; (b) active surveillance for illness by the CHWs during routine postnatal home-visits; (c) facilitated referral by the CHWs, including counselling, use of referral slips along with improved linkages between community and hospital; (d) incentives for labour/birth notification; (e) enhanced capacity at the referral-care centre to manage sick newborns; and (f) availability of subsidized treatment.  Sustained community-level education enhanced the empowerment of families towards decision-making for self-referral. | Fair |
| **Criterion #9**  **Description of materials:** Description of all materials or tools used for the implementation  (From CReDECI, Mohler, 2012)  **Explanation/Example:**  "The primary enablers of behaviour change were paid community-based health workers, who were recruited from the local community based on 12 years or more of education,  proficient communication and reasoning skills, commitment towards community work, and references of community stakeholders. They received a combination of classroombased  and apprentice ship-based field training over 7 days on knowledge, attitudes, and practices related to essential newborn care within the community, behaviour change management, and trust-building. After training, suitable candidates were closely mentored and supervised by a regional programme supervisor (n=4) responsible for 6–7 trainees, for an additional week before final selection was made." | No text found | Poor |
| **Criterion #10**  **Process of Implementation:** Description of an assessment of the implementation process  (From CReDECI, Mohler 2012)  **Explanation/Example:**  Process assessment is a prerequisite for determining the success of the intervention's implementation and should be an integral part of an assessment of the intervention’s effect. For example, "To gain insight into the dissemination and the delivery of the  intervention and to draw conclusions about potential barriers and facilitators to implementing the intervention in other settings, data on the implementation process were collected alongside the randomized-controlled trial. Therefore, we assessed the quality of  delivery of the interventional components (observed by members of the research team not involved in the delivery of the intervention) and the adherence to study protocol (number  and type of deviations from the protocol, using a pilot-tested standardized form). We also analyzed barriers and facilitators for the delivery of intervention’s components (focus  group interviews with intervention participants)." | After three months of initial implementation of the programme, further changes were made to improve coverage with home-visits and compliance with referrals. A referral-tracking form was introduced in April 2004,  and every two weeks, the number of newborns referred and the outcome of referral were reviewed in a meeting with the supervisors and CHWs. In early 2005, a decision was made to emphasize more the management and referral of birth asphyxia and low-birth-weight newborns. The CHWs started to use digital weighing machines in February 2005 to obtain the weight of the  newborn at first contact. In April 2005, the CHWs received refresher training on how to counsel families during antenatal home-visits on low birth-weight and birth asphyxia. | Good |

**Additional File 2-M. Criterion Table for Chongsuvivatwong et al.; 1996**

| **Criterion** | **Example of text related to this criterion** | **Rating** |
| --- | --- | --- |
| **Criterion #1**  **Intervention Characteristics:** Intervention/Program source  (From CFIR, Damschroder, 2009)  **Explanation/Example:**  Is the intervention/program externally or internally developed? An intervention/program may be internally developed as a good idea, a solution to a problem, or other grass roots effort, or may be developed by an external entity (such as a foundation or a NGO). Interventions or programs that arise internally from the populations who will be impacted are sometimes more sustainable than externally developed programs dependent on external funding. The perceived legitimacy of the source may also influence implementation. | In the early 1990s, Thailand spent approximately 4 million US dollars in 5 years, educating pediatricians, doctors in district hospitals, health workers in health centers and village health volunteers with the aim of improving case detection and case management. The current study aimed to assess the impact of the training program on change of health care utilization and change of average sick days among the affected children. (States intervention was externally developed in government)  The curriculum and media for training were developed and distributed by the Ministry of Public Health in Bangkok. | Good |
| **Criterion #2**  **Intervention Characteristics:** A description of why the intervention was hypothesized to have an impact on the outcome, according to theory. (From CReDECI, Mohler 2012; also mentioned in Michie, 2009)  **Explanation/Example:**  The theoretical basis of the intervention should be clearly stated. This includes the theory on which the intervention is founded as well as, if available, empirical evidence from studies in different settings or countries. For example, "The implementation was based on Rogers’ Diffusion of Innovation theory, which posits 5 factors of innovation that influence a decision to adopt or reject an innovation: relative advantage, compatibility, complexity or simplicity, trialability, observability. A similar intervention, also based on Rogers’ Diffusion of Innovation theory, was successfully implemented in other countries." | No text found | Poor |
| **Criterion #3**  **Intervention Characteristics:**  Rationale for the aim/essential functions of the intervention/program’s components, including the evidence whether the components are appropriate for achieving this goal.  This differs from the need to articulate the theory behind the intervention in that the theory posits the general principles (such as Rogers Diffusion of Innovation) while this item is about specific components of the intervention and the effects of the component on specific targets.  (From CReDECI, Mohler, 2012; also mentioned in Michie, 2009) | No text found | Poor |
| **Criterion #4**  **Intervention Characteristics:**  Detailed description of the intervention/program (From WIDER as described in Michie, 2009)  **The detailed description should include:**  a. Characteristics of those delivering the intervention/program (such as a nurse or lay health worker)  b. Characteristics of the recipients  c. The setting  d. The mode of delivery (such as face-to-face)  e. The intensity of the intervention/program (such as the contact time with participants)  f. The duration (such as the number of sessions and their spacing interval over a given period)  g. Adherence or fidelity to delivery protocols  h. A detailed description of the intervention/program content provided to each study group | a. The health workers who had been trained by pediatricians at the regional hospital collectively conducted a 2-day training workshop for the study villages. Approximately 10 health volunteers from each of the study villages participated in the workshop.  b. Table 1 provides some data about participants  c. The study was conducted from January to May 1991 in Nong Chik District, Pattani Province. The community has a low socioeconomic status. The total population is approximately 50,000. The district is administratively divided into 12 Tumbons (or subdistricts) comprising all together 67 villages. The expected number of 0-5 year old population was between 4,000 and 5,000. There is one 10-bed hospital with one doctor and 13 health centers, each with two or more health workers (a midwife and a sanitarian). The 200-bed provincial hospital is 15km from the community. Buses from all villages to these hospitals were available at least once a day.  d. Two weeks after the training workshop, all the children under 5 years old in the 30 villages were visited by a team of home visitors once a week for a maximum of 19 weeks.  e. Two weeks after the training workshop, all the children under 5 years old in the 30 villages were visited by a team of home visitors once a week for a maximum of 19 weeks.  f. Two weeks after the training workshop, all the children under 5 years old in the 30 villages were visited by a team of home visitors once a week for a maximum of 19 weeks.  g. No text found  h. No text found | Fair  Fair  Good  Good  Fair  Good  Poor  Poor |
| **Criterion #5**  **Intervention Characteristics:**  Costs of the intervention and costs associated with implementing the intervention (From CFIR, Damschroder, 2009; CReDECI, Mohler, 2012)  **Explanation/Example:**  The cost of the intervention and implementation can influence the adoption and sustainability; interventions maybe more difficult to sustain if they were supported as part of a research study. | No text found | Poor |
| **Criterion #6**  **Outer Setting:** External policies and incentives (From CFIR, Damschroder, 2009)  **Explanation/Example:**  How does the health service, intervention, or program relate to country and global health goals? Is the program part of a larger strategy? If so how is it strategically aligned? A country's health policies may influence the implementation of a particular intervention or program. | In the early 1990s, Thailand spent approximately 4 million US dollars in 5 years, educating pediatricians, doctors in district hospitals, health workers in health centers and village health volunteers with the aim of  improving case detection and case management. | Good |
| **Criterion #7**  **Population needs**  (From CFIR, Damschroder, 2009)  **Explanation/Example:**  The extent to which population needs, as well as barriers and facilitators to meet those needs, are accurately known and prioritized. This could include population-based data on causes of morbidity and mortality, political or cultural barriers or facilitators, and/or more locally focused data about local needs, barriers or facilitators. | In Thailand, health statistics show that ARI is the most common cause of death among children (Ministry of Public Health, 1995). | Fair |
| **Criterion #8**  **Process of implementation:** Description of facilitators or barriers which have influenced the intervention or program’s implementation (see #10) revealed by a process assessment.  In contrast to the criterion #7 above which assesses barriers and facilitators as inputs to developing the intervention strategy, this criterion assesses the actual barriers and facilitators identified during and after the implementation.  (From CReDECI, Mohler, 2012; also mentioned in Michie, 2009)  **Explanation/Example:**  "The attitudes of the nursing home managers turned out to be an important factor supporting or impeding the success of the intervention's implementation. The more the managers agreed with the interventions’ aim, the better the nursing staff felt supported." | No text found | Poor |
| **Criterion #9**  **Description of materials:** Description of all materials or tools used for the implementation  (From CReDECI, Mohler, 2012)  **Explanation/Example:**  "The primary enablers of behaviour change were paid community-based health workers, who were recruited from the local community based on 12 years or more of education,  proficient communication and reasoning skills, commitment towards community work, and references of community stakeholders. They received a combination of classroombased  and apprentice ship-based field training over 7 days on knowledge, attitudes, and practices related to essential newborn care within the community, behaviour change management, and trust-building. After training, suitable candidates were closely mentored and supervised by a regional programme supervisor (n=4) responsible for 6–7 trainees, for an additional week before final selection was made." | No text found | Poor |
| **Criterion #10**  **Process of Implementation:** Description of an assessment of the implementation process  (From CReDECI, Mohler 2012)  **Explanation/Example:**  Process assessment is a prerequisite for determining the success of the intervention's implementation and should be an integral part of an assessment of the intervention’s effect. For example, "To gain insight into the dissemination and the delivery of the  intervention and to draw conclusions about potential barriers and facilitators to implementing the intervention in other settings, data on the implementation process were collected alongside the randomized-controlled trial. Therefore, we assessed the quality of  delivery of the interventional components (observed by members of the research team not involved in the delivery of the intervention) and the adherence to study protocol (number  and type of deviations from the protocol, using a pilot-tested standardized form). We also analyzed barriers and facilitators for the delivery of intervention’s components (focus  group interviews with intervention participants)." | No text found | Poor |

**Additional File 2-N. Criterion Table for Manandhar et al.; 2004**

| **Criterion** | **Example of text related to this criterion** | **Rating** |
| --- | --- | --- |
| **Criterion #1**  **Intervention Characteristics:** Intervention/Program source  (From CFIR, Damschroder, 2009)  **Explanation/Example:**  Is the intervention/program externally or internally developed? An intervention/program may be internally developed as a good idea, a solution to a problem, or other grass roots effort, or may be developed by an external entity (such as a foundation or a NGO). Interventions or programs that arise internally from the populations who will be impacted are sometimes more sustainable than externally developed programs dependent on external funding. The perceived legitimacy of the source may also influence implementation. | Previously, we showed no effect of direct education by health workers on infant care practices and care-seeking behaviour after delivery. In view of the Bolivian model, we thought that a participatory approach might have more effect on perinatal care practices and might increase consultation for difficulties in pregnancy and the newborn period. (implies that the intervention idea was external) | Good |
| **Criterion #2**  **Intervention Characteristics:** A description of why the intervention was hypothesized to have an impact on the outcome, according to theory. (From CReDECI, Mohler 2012; also mentioned in Michie, 2009)  **Explanation/Example:**  The theoretical basis of the intervention should be clearly stated. This includes the theory on which the intervention is founded as well as, if available, empirical evidence from studies in different settings or countries. For example, "The implementation was based on Rogers’ Diffusion of Innovation theory, which posits 5 factors of innovation that influence a decision to adopt or reject an innovation: relative advantage, compatibility, complexity or simplicity, trialability, observability. A similar intervention, also based on Rogers’ Diffusion of Innovation theory, was successfully implemented in other countries." | Previously, we showed no effect of direct education by health workers on infant care practices and care-seeking behaviour after delivery. In view of the Bolivian model, we thought that a participatory approach might have more effect on perinatal care practices and might increase consultation for difficulties in pregnancy and the newborn period.  Two key elements distinguished our approach from conventional health education. First, women’s groups looked at demand-side and supply-side issues. Second, the approach emphasised participatory learning rather than instruction. | Good |
| **Criterion #3**  **Intervention Characteristics:**  Rationale for the aim/essential functions of the intervention/program’s components, including the evidence whether the components are appropriate for achieving this goal.  This differs from the need to articulate the theory behind the intervention in that the theory posits the general principles (such as Rogers Diffusion of Innovation) while this item is about specific components of the intervention and the effects of the component on specific targets.  (From CReDECI, Mohler, 2012; also mentioned in Michie, 2009) | The intervention needed a facilitator rather than a teacher, with abilities and training in participatory communication techniques.  The first step of the intervention was to discuss issues around childbirth and care behaviours in the community, which allowed facilitators to develop participatory learning skills and generated information on pregnancy and childbirth, covering beliefs and practices in both uncomplicated and complicated pregnancies.  A baseline service audit identified weaknesses in the provision of antenatal, delivery, and newborn care in Makwanpur district. | Fair |
| **Criterion #4**  **Intervention Characteristics:**  Detailed description of the intervention/program (From WIDER as described in Michie, 2009)  **The detailed description should include:**  a. Characteristics of those delivering the intervention/program (such as a nurse or lay health worker)  b. Characteristics of the recipients  c. The setting  d. The mode of delivery (such as face-to-face)  e. The intensity of the intervention/program (such as the contact time with participants)  f. The duration (such as the number of sessions and their spacing interval over a given period)  g. Adherence or fidelity to delivery protocols  h. A detailed description of the intervention/program content provided to each study group | a. For every intervention cluster we recruited one local female facilitator. Shortlistsor this role were derived from nomination by community leaders, advertisement, and word of mouth, after which all potential candidates were interviewed.  The facilitator—a literate locally resident woman—convened one women’s group meeting a month in every ward (figure 2).  b. All 43 village development committees in Makwanpur district were eligible for randomisation, of which one was excluded at baseline for security reasons. We enrolled a closed cohort of married women of reproductive age. Inclusion criteria were: consent given for involvement; age 15–49 years inclusive on June 15, 2000; married; and potential to become pregnant. Exclusion criteria included long-term separation from spouse and widowhood. Women who  chose to participate in the study gave verbal consent and were free to decline to be interviewed at any time.  In addition, Table 2 presents data on the recipients.  c. With a population of more than 23 million and a gross national income of US$240 per person,20 Nepal is a poor country whose development challenges are exacerbated by its geography and unstable political situation. Life expectancy is 61 years. The total fertility rate is 4·4 children per woman in rural areas,21 and the estimated maternal mortality ratio is 539 per 100 000 livebirths.22 57% of women cannot read.23 The estimated infant mortality rate is 64 per 1000 live births, the neonatal mortality rate 39 per 1000 live births, and the perinatal mortality rate 47 per 1000 births.21 In rural areas, 94% of babies are born at home,22 and only 13% of births are attended by trained health workers.  d. Makwanpur district lies in Nepal’s central region where the middle hills join the plains. The population of about 400 000 subsists mainly on agriculture and the largest ethnic groups are Tamang and Brahmin-Chhetri. The district hospital in the municipality of Hetauda has facilities for antenatal care and delivery, although operative delivery was not available during the study period. There are 7852 people per hospital bed.24 The district health system makes perinatal care available through a network of primary health centres, health posts, subhealth posts, and outreach clinics. Traditional birth attendants are available throughout the district, but their attendance at births is less common than in some other parts of south Asia.  e. Health-service strengthening activities were undertaken in both intervention and control areas. (This is stated as face-to-face)  f. The cluster interviewer did two interviews for every pregnancy: the first at 7 months of gestation, as near as possible to the transition between our definitions of miscarriage and stillbirth; and the second at 1 month postpartum, as near as possible to the transition between neonatal and infancy periods.  The facilitators then supported the women’s groups through monthly meetings (table 1). This phase of ten meetings lasted almost a year.  g. All 24 clusters selected for inclusion received their allocated intervention.  h. No text found | Good  Good  Good  Good  Good  Good  Fair  Poor |
| **Criterion #5**  **Intervention Characteristics:**  Costs of the intervention and costs associated with implementing the intervention (From CFIR, Damschroder, 2009; CReDECI, Mohler, 2012)  **Explanation/Example:**  The cost of the intervention and implementation can influence the adoption and sustainability; interventions maybe more difficult to sustain if they were supported as part of a research study. | A cost-effectiveness analysis was done alongside the study. The cost per newborn life saved was US$3442 ($4397 including health-service strengthening costs) and  per life year saved $111 ($142 including health-service strengthening costs). | Fair |
| **Criterion #6**  **Outer Setting:** External policies and incentives (From CFIR, Damschroder, 2009)  **Explanation/Example:**  How does the health service, intervention, or program relate to country and global health goals? Is the program part of a larger strategy? If so how is it strategically aligned? A country's health policies may influence the implementation of a particular intervention or program. | No text found | Poor |
| **Criterion #7**  **Population needs**  (From CFIR, Damschroder, 2009)  **Explanation/Example:**  The extent to which population needs, as well as barriers and facilitators to meet those needs, are accurately known and prioritized. This could include population-based data on causes of morbidity and mortality, political or cultural barriers or facilitators, and/or more locally focused data about local needs, barriers or facilitators. | The estimated infant mortality rate is 64 per 1000 live births, the neonatal mortality rate 39 per 1000 live births, and the perinatal mortality rate 47 per 1000 births.21 In rural areas, 94% of babies are born at home,22 and only 13% of births are attended by trained health workers. | Good |
| **Criterion #8**  **Process of implementation:** Description of facilitators or barriers which have influenced the intervention or program’s implementation (see #10) revealed by a process assessment.  In contrast to the criterion #7 above which assesses barriers and facilitators as inputs to developing the intervention strategy, this criterion assesses the actual barriers and facilitators identified during and after the implementation.  (From CReDECI, Mohler, 2012; also mentioned in Michie, 2009)  **Explanation/Example:**  "The attitudes of the nursing home managers turned out to be an important factor supporting or impeding the success of the intervention's implementation. The more the managers agreed with the interventions’ aim, the better the nursing staff felt supported." | No text found | Poor |
| **Criterion #9**  **Description of materials:** Description of all materials or tools used for the implementation  (From CReDECI, Mohler, 2012)  **Explanation/Example:**  "The primary enablers of behaviour change were paid community-based health workers, who were recruited from the local community based on 12 years or more of education,  proficient communication and reasoning skills, commitment towards community work, and references of community stakeholders. They received a combination of classroom based and apprentice ship-based field training over 7 days on knowledge, attitudes, and practices related to essential newborn care within the community, behaviour change management, and trust-building. After training, suitable candidates were closely mentored and supervised by a regional programme supervisor (n=4) responsible for 6–7 trainees, for an additional week before final selection was made." | No text found | Poor |
| **Criterion #10**  **Process of Implementation:** Description of an assessment of the implementation process  (From CReDECI, Mohler 2012)  **Explanation/Example:**  Process assessment is a prerequisite for determining the success of the intervention's implementation and should be an integral part of an assessment of the intervention’s effect. For example, "To gain insight into the dissemination and the delivery of the  intervention and to draw conclusions about potential barriers and facilitators to implementing the intervention in other settings, data on the implementation process were collected alongside the randomized-controlled trial. Therefore, we assessed the quality of  delivery of the interventional components (observed by members of the research team not involved in the delivery of the intervention) and the adherence to study protocol (number  and type of deviations from the protocol, using a pilot-tested standardized form). We also analyzed barriers and facilitators for the delivery of intervention’s components (focus  group interviews with intervention participants)." | No text found | Poor |

**Additional File 2-O. Criterion Table for Marsh et al.; 2002**

| **Criterion** | **Example of text related to this criterion** | **Rating** |
| --- | --- | --- |
| **Criterion #1**  **Intervention Characteristics:** Intervention/Program source  (From CFIR, Damschroder, 2009)  **Explanation/Example:**  Is the intervention/program externally or internally developed? An intervention/program may be internally developed as a good idea, a solution to a problem, or other grass roots effort, or may be developed by an external entity (such as a foundation or a NGO). Interventions or programs that arise internally from the populations who will be impacted are sometimes more sustainable than externally developed programs dependent on external funding. The perceived legitimacy of the source may also influence implementation. | The ViSION project involved a partnership among Save the Children/US ([SC] Hanoi and Westport, Conn., USA), the USAID-funded LINKAGE Project (Washington, D.C.), Emory University Rollins School of Public Health (Atlanta, Ga., USA), and the Research and Training Center for Community Development (RTCCD, Hanoi). The SC Viet Nam field office developed the program model and implemented it through government partners. (Indicates this is externally funded) | Good |
| **Criterion #2**  **Intervention Characteristics:** A description of why the intervention was hypothesized to have an impact on the outcome, according to theory. (From CReDECI, Mohler 2012; also mentioned in Michie, 2009)  **Explanation/Example:**  The theoretical basis of the intervention should be clearly stated. This includes the theory on which the intervention is founded as well as, if available, empirical evidence from studies in different settings or countries. For example, "The implementation was based on Rogers’ Diffusion of Innovation theory, which posits 5 factors of innovation that influence a decision to adopt or reject an innovation: relative advantage, compatibility, complexity or simplicity, trialability, observability. A similar intervention, also based on Rogers’ Diffusion of Innovation theory, was successfully implemented in other countries." | In brief, the intervention was based on the positive deviant approach, in which foods and caregiver behaviors of better-nourished children from resource poor settings were identified and incorporated into the nutritional intervention program. | Good |
| **Criterion #3**  **Intervention Characteristics:**  Rationale for the aim/essential functions of the intervention/program’s components, including the evidence whether the components are appropriate for achieving this goal.  This differs from the need to articulate the theory behind the intervention in that the theory posits the general principles (such as Rogers Diffusion of Innovation) while this item is about specific components of the intervention and the effects of the component on specific targets.  (From CReDECI, Mohler, 2012; also mentioned in Michie, 2009) | A separate publication devoted to the design of the study spends four pages of text detailing the different components and their intended effect | Good |
| **Criterion #4**  **Intervention Characteristics:**  Detailed description of the intervention/program (From WIDER as described in Michie, 2009)  **The detailed description should include:**  a. Characteristics of those delivering the intervention/program (such as a nurse or lay health worker)  b. Characteristics of the recipients  c. The setting  d. The mode of delivery (such as face-to-face)  e. The intensity of the intervention/program (such as the contact time with participants)  f. The duration (such as the number of sessions and their spacing interval over a given period)  g. Adherence or fidelity to delivery protocols  h. A detailed description of the intervention/program content provided to each study group | a. Only described as “local health volunteers” or “health volunteers” or “district trainers”  b. Table 1 lists data on characteristics of the participants  c. The study took place in Phu Tho Province, 98 km northwest of Hanoi. This north central ecological region has the worst child nutrition profile (47% underweight, 46.5% stunted, and 9.9% wasted) in the country.* The province has 1.3 million rural lowland, midland, and highland inhabitants in thousands of hamlets in 249 communes in eight districts. The main ethnic group is Kinh, the predominant national majority, with some Muong, Dao, San Chay, and San Diu minorities. The population is poor with an average per capita monthly rice production of 17.8 kg [and it goes on for another three paragraphs and table describes setting].  d. There are multiple different components, but most or all interaction are face-to-face.  They conducted the PDI with health volunteers by visiting the six families and using a question and observation guide to gather information from family members about child feeding, care, and health. The CENP teams also visited the better-off families with malnourished children to identify the causes of their children’s malnutrition and to highlight the lesson that money was neither necessary nor sufficient for child health. District trainers attempted to schedule the PDI to allow observation of a child feeding. They followed a question guide with prompts and took notes. Accompanying health volunteers were encouraged to ask questions. The CENP team and interested community members convened in an open forum to review the cases and to develop a summary profile for their commune of uncommon behaviors that seemed to contribute to the positive deviant (PD) children’s good nutritional status despite their families’ poverty. The team invited community input to develop a final consensus. the intervention proper consisted of GMP sessions every two months, monthly NERP sessions as long as the number of malnourished children warranted them for up to nine months. All channel B, C, and D children, i.e., less than –2 WAZ, were referred to NERPs. Health volunteers conducted monthly NERP sessions for 12 days (six days weekly for two weeks) in their hamlets to enable families to both rehabilitate and to sustain the enhanced nutritional status of their malnourished children. On each of the 12 NERP days each participating child received a nutritious meal prepared by two or more caregivers in rotation. The meal was designed as additional to the usual diet, but in practice it may have substituted for another meal [5]. Health volunteers allowed mothers to take home the unconsumed food if the child was reluctant to eat at the NERP session.  In addition, Table 4 in Marsh lists intensity information.  f. 9 months  g. A concomitant process evaluation assessed adherence to protocol (results reported in table 3).  h.SC staff trained district trainers and members of the CMSC in the concepts and methods of the positive deviance approach using the CENP training manual developed by SC. | Poor  Good  Good  Good  Good  Good  Good  Good |
| **Criterion #5**  **Intervention Characteristics:**  Costs of the intervention and costs associated with implementing the intervention (From CFIR, Damschroder, 2009; CReDECI, Mohler, 2012)  **Explanation/Example:**  The cost of the intervention and implementation can influence the adoption and sustainability; interventions maybe more difficult to sustain if they were supported as part of a research study. | Total program cost was approximately US$ 12,000 for the six communes. (Not clear if this is total cost or just sponsor costs) | Fair |
| **Criterion #6**  **Outer Setting:** External policies and incentives (From CFIR, Damschroder, 2009)  **Explanation/Example:**  How does the health service, intervention, or program relate to country and global health goals? Is the program part of a larger strategy? If so how is it strategically aligned? A country's health policies may influence the implementation of a particular intervention or program. | No text found | Poor |
| **Criterion #7**  **Population needs**  (From CFIR, Damschroder, 2009)  **Explanation/Example:**  The extent to which population needs, as well as barriers and facilitators to meet those needs, are accurately known and prioritized. This could include population-based data on causes of morbidity and mortality, political or cultural barriers or facilitators, and/or more locally focused data about local needs, barriers or facilitators. | A long list of community health and nutrition status variables are presented | Good |
| **Criterion #8**  **Process of implementation:** Description of facilitators or barriers which have influenced the intervention or program’s implementation (see #10) revealed by a process assessment.  In contrast to the criterion #7 above which assesses barriers and facilitators as inputs to developing the intervention strategy, this criterion assesses the actual barriers and facilitators identified during and after the implementation.  (From CReDECI, Mohler, 2012; also mentioned in Michie, 2009)  **Explanation/Example:**  "The attitudes of the nursing home managers turned out to be an important factor supporting or impeding the success of the intervention's implementation. The more the managers agreed with the interventions’ aim, the better the nursing staff felt supported." | **Implementation**  SC succeeded in teaching district Ministry of Health partners to train local implementers to conduct a complex set of interventions: GMP, PDI, and NERP. Despite the implementers’ characteristic enthusiasm, some field realities prompt caution. The CENP is designed as a rehabilitation model for lowland, densely populated communes with high levels of severe malnutrition.  Neither condition prevailed in the selected districts, which were drawn from a pool of similar districts with minimal non-governmental organizational activity  and a SC commitment to introduce the program. Political support in Phu Tho province for district and commune selection was more restrained than in earlier iterations. These communes also had baseline levels of malnutrition that were far less than officially reported. In response, SC recommended including moderately malnourished children in the NERPs and revised  graduation criteria, a modification that had been contemplated,  but not tested. Thus, compared to children in prior CENPs, these intervention children were more likely to be enrolled in and slower to graduate from NERPs. Meanwhile, NERP implementation deviated from protocol in that daily contributions were not the norm, and home-delivered meals were common, perhaps because the population was dispersed, or caregivers were less concerned about moderate malnutrition than they would have been about severe malnutrition. It is possible that this and other local adaptations may have been implemented earlier in other settings without the knowledge of SC. | Good |
| **Criterion #9**  **Description of materials:** Description of all materials or tools used for the implementation  (From CReDECI, Mohler, 2012)  **Explanation/Example:**  "The primary enablers of behaviour change were paid community-based health workers, who were recruited from the local community based on 12 years or more of education, proficient communication and reasoning skills, commitment towards community work, and references of community stakeholders. They received a combination of classroom based and apprentice ship-based field training over 7 days on knowledge, attitudes, and practices related to essential newborn care within the community, behaviour change management, and trust-building. After training, suitable candidates were closely mentored and supervised by a regional programme supervisor (n=4) responsible for 6–7 trainees, for an additional week before final selection was made." | The publication references a five volume field training manual | Good |
| **Criterion #10**  **Process of Implementation:** Description of an assessment of the implementation process  (From CReDECI, Mohler 2012)  **Explanation/Example:**  Process assessment is a prerequisite for determining the success of the intervention's implementation and should be an integral part of an assessment of the intervention’s effect. For example, "To gain insight into the dissemination and the delivery of the intervention and to draw conclusions about potential barriers and facilitators to implementing the intervention in other settings, data on the implementation process were collected alongside the randomized-controlled trial. Therefore, we assessed the quality of delivery of the interventional components (observed by members of the research team not involved in the delivery of the intervention) and the adherence to study protocol (number and type of deviations from the protocol, using a pilot-tested standardized form). We also analyzed barriers and facilitators for the delivery of intervention’s components (focus group interviews with intervention participants)." | In summary, this evaluation documents the difference between CENP protocol and implementation and the effectiveness of the implementation in a challenging field setting. While the implementation may be similar to previous iterations, the effectiveness may be somewhat less, given the challenging field conditions and changes to protocol. Our thorough understanding of the CENP implementation allows us to better interpret its effect and thus the potential of PD-informed programs. | Good |
